# Structure–efficiency relationships of cyclodextrin scavengers in the hydrolytic degradation of organophosphorus compounds

**DOI:** 10.3762/bjoc.13.45

**Published:** 2017-03-06

**Authors:** Sophie Letort, Michaël Bosco, Benedetta Cornelio, Frédérique Brégier, Sébastien Daulon, Géraldine Gouhier, François Estour

**Affiliations:** 1 Normandie Univ, INSA Rouen, UNIROUEN, CNRS, COBRA (UMR 6014), 76000 Rouen, France; 2 DGA Maîtrise NRBC, Département Evaluation des effets des agents chimiques, 91710 Vert le Petit, France

**Keywords:** cyclodextrin, decontamination, enzyme mimic, nerve agents, organophosphorus pesticides

## Abstract

New derivatives of cyclodextrins were prepared in order to determine the relative importance of the structural key elements involved in the degradation of organophosphorus nerve agents. To avoid a competitive inclusion between the organophosphorus substrate and the iodosobenzoate group, responsible for its degradation, the latter group had to be covalently bound to the cyclodextrin scaffold. Although the presence of the α nucleophile iodosobenzoate was a determinant in the hydrolysis process, an imidazole group was added to get a synergistic effect towards the degradation of the agents. The degradation efficiency was found to be dependent on the relative position of the heterocycle towards the reactive group as well as on the nature of the organophosphorus derivative.

## Introduction

Originally employed as pesticides, organophosphorus compounds were further developed as chemical warfare agents during the Second World War. These compounds act as potent irreversible inhibitors of cholinesterases [1–6] and are able to cause lethal intoxications [3]. Despite the measures adopted to reduce the risk of accidental poisoning by pesticides [7–11] and the Chemical Weapons Convention aiming at the non-proliferation of chemical weapons or their precursors, organophosphorus compounds still constitute a threat to civilian and military people. Moreover, due to the current geopolitical situation and the increasing number of terrorist attacks worldwide, more efficient means against nerve agents are required [12]. Four steps have to be considered to reach this objective: detection, individual and collective protection, decontamination, and medical countermeasures. Because a contamination transfer can occur from victims or through contact with contaminated equipment, a rapid elimination of the toxic has to be envisaged. For this, a scavenging approach to trap and degrade the nerve agents seems especially promising and may consist in developing enzyme mimics able to hydrolyze the organophosphorus (OP) compounds under physiological conditions.

**Figure 1:**
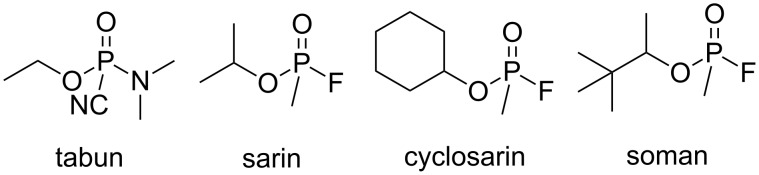
Structures of G agents.

In this context, cyclodextrins (CD) constitute attractive starting materials because, due to the inclusion properties of their internal cavity, they can form host–guest complexes in aqueous media by weak interactions with small hydrophobic molecules. In particular, these macromolecular structures display the interesting capability to include organophosphorus pesticides into their cavity [13–17]. However, their intrinsic ability to transform these compounds into low or non-toxic metabolites at physiological pH is weak [18–20]. Therefore, in order to display such metabolic efficiency under mild conditions, various monofunctionalization strategies of β-CD were studied [21–22]. The attachment of an α-nucleophilic functional group on β-CD is a promising strategy to degrade G agents such as soman, sarin, cyclosarin or tabun (Figure 1) [23–30]. In fact, these β-CD derivatives play a dual role in this process: the macrocycle traps the organophosphorus whilst the bound α nucleophile reacts with the toxic agent leading to a non-toxic derivative. Other scavengers bearing several α nucleophilic groups were described [31–32].

**Figure 2:**
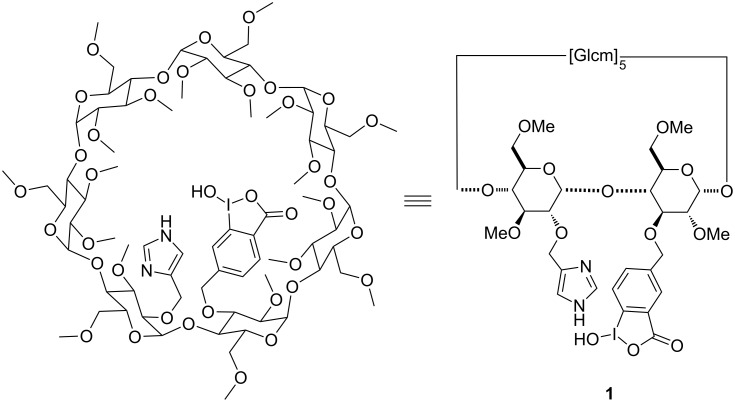
Scavenger based on a heterodifunctionalized β-cyclodextrin derivative.

Recently, our team developed a synthesis of heterodifunctionalized β-CD derivatives bearing an iodosobenzoate group and an imidazole substituent [33]. We have proven that the presence of both substituents increased the detoxification rate of soman as compared to the monofunctionalized derivatives. However, the synergistic effect was regiodependent and only observed with the imidazole substituent located in position 2 of one methylated glucose unit and the α nucleophile in position 3 of the adjacent methylated glucose unit (compound **1**, Figure 2).

**Figure 3:**
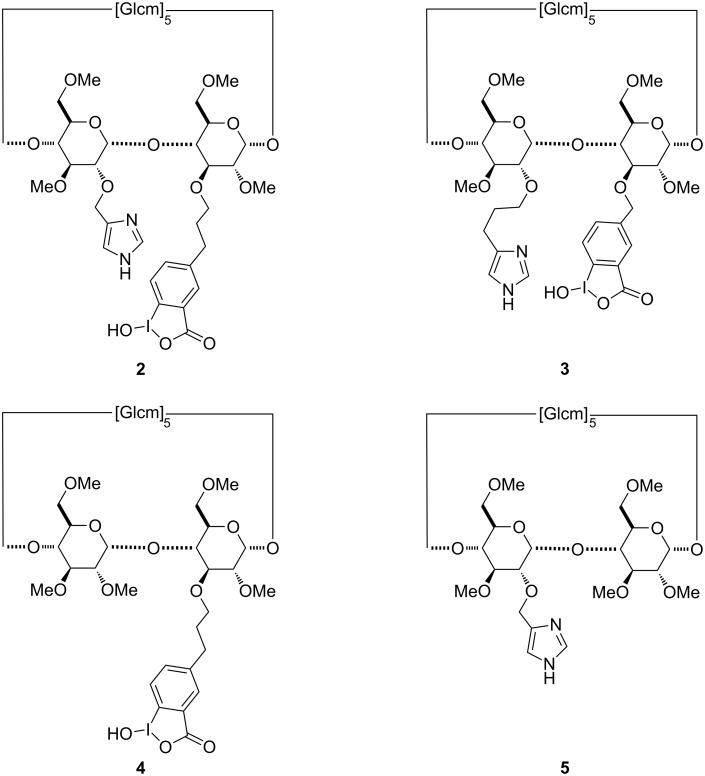
Structures of β-cyclodextrin derivatives **2**–**5**.

Herein we present an extended study focusing on the impact of covalently bound functional groups to macrocyclic β-cyclodextrin that are involved in the OP hydrolysis. Four new derivatives **2–5** were prepared (Figure 3) for this purpose. Compared to analog **1**, scavenger **2** has a longer linker between the iodosobenzoate group and the methylated-β-cyclodextrin scaffold whilst scavenger **3** is characterized by a longer linker binding the imidazole ring to the CD derivative. Finally, compounds **4** and **5** are analogs of **2** bearing only one of these groups, either the α nucleophile or the imidazole ring, respectively.

**Figure 4:**
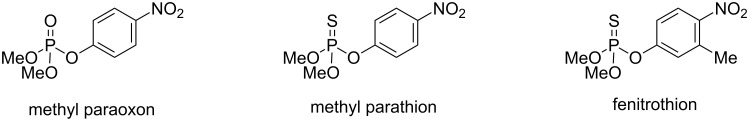
Structures of pesticides tested.

All five derivatives **1**–**5** were tested for their degradation ability against methyl paraoxon (Figure 4), selected as the pesticide model, and their efficiencies were compared. To demonstrate the importance of functionalizing the CDs and the influence of the individual moieties, the experiments were performed using the modified scavengers (with the groups covalently attached to the macrocycle) and with mixtures of heptakis(2,3,6-trimethyl)-β-cyclodextrin (TRIMEB) with 2-iodosobenzoic acid and/or imidazole, respectively. In addition, the degradation properties of the newly synthesized CD derivatives against methyl parathion and fenitrothion (Figure 4) were also investigated. Finally, compounds **1**–**4** were tested for their detoxification ability against the nerve agent soman.

## Results and Discussion

### Synthesis

**Scheme 1:**
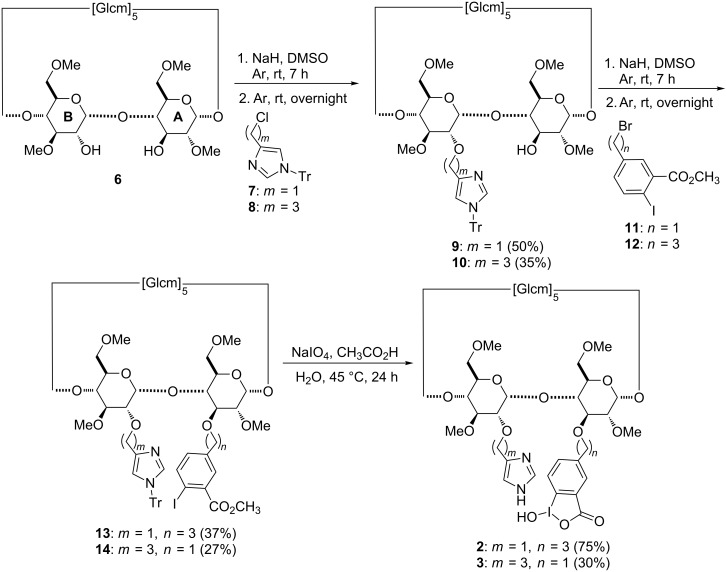
Synthetic pathway to derivatives **2** and **3** (Tr = trityl).

The regioselective disubstitution of diol **6** (Scheme 1) was the key step to access derivatives **2** and **3**.

The synthetic methodology consisted first in the selective introduction of the imidazole substituent in position 2 in unit B of **6** by making use of the higher acidity of this hydroxy group compared to the OH groups in positions 3 and 6. As expected, the substitution reaction with the benzyl-like reactant **7** led to a higher yield than the reaction with the propyl analog **8** (50% versus 35%), but suffered from a slightly lower regioselectivity. In fact, 4% of the 3-monosubstituted regioisomer of **9** was also formed, whereas less than 1% of the 3-monofunctionalized regioisomer of **10** was observed for the reaction with electrophile **8**. Once the first group was introduced in position 2, the substitution reaction at O-3 on the adjacent unit A was performed. Due to the lower reactivity of this alcohol group, an excess of base and electrophile was required for this step. In addition, the presence of the sterically hindered trityl-protected imidazole reduced the accessibility of substrates **11** and **12** to position 3. Thus, compounds **13** and **14** were isolated in comparable yields of 37% and 27%, respectively and this time without significantly different reactivities observed for the precursors **9** and **10**.

High resolution mass spectrometry (ESI^+^ HRMS) analyses of compounds **13** and **14** confirmed the presence of the two substituents bound to the macrocycle. Also the ^1^H and ^13^C NMR spectra showed chemical shifts attributable to the CH_2_ groups linked to the imidazole and iodobenzoate moieties, similar to those observed for the precursor of **1** [33]. The subsequent deprotection and oxidation–hydrolysis reactions of derivatives **13** and **14** afforded scavengers **2** and **3**, respectively and were performed in one step using sodium periodate in acidic medium. Compound **2** was obtained in good yield, but the steric hindrance of the benzylic group in derivative **14** decreased the yield of scavenger **3**.

**Scheme 2:**
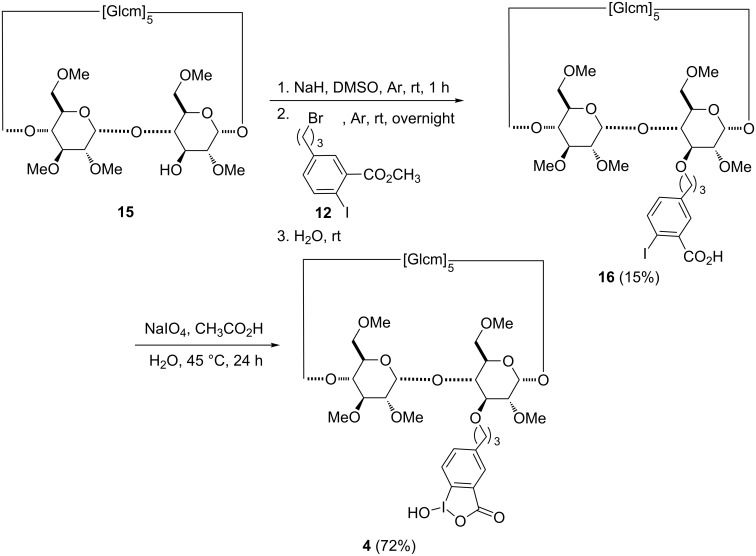
Synthesis of compound **4**.

The introduction of the methyl iodobenzoate substituent at O-3 was conducted starting from monohydroxy compound **15** [34]. After reaction with electrophile **12**, compound **4** was obtained through oxidation and hydrolysis of intermediate **16** (Scheme 2) using the same experimental conditions as applied for derivatives **13** and **14**. HSQC NMR analysis of **16** allowed the assignment of the three CH_2_ groups of the propyl linker bearing the benzene ring. Correlation signals were observed for the diastereotopic protons at 3.59 and 3.90 ppm with the ^13^C signal at 73.1 ppm belonging to the carbon connected to the oxygen atom at the C-3 position. In the same way, the HSQC spectrum highlights a correlation between two other diastereotopic protons 2.50 and 2.75 ppm) and the benzylic carbon (31.9 ppm). Finally, the quintuplet at 1.99 ppm correlated with the ^13^C signal at 31.4 ppm was assigned to the third methylene group.

**Scheme 3:**
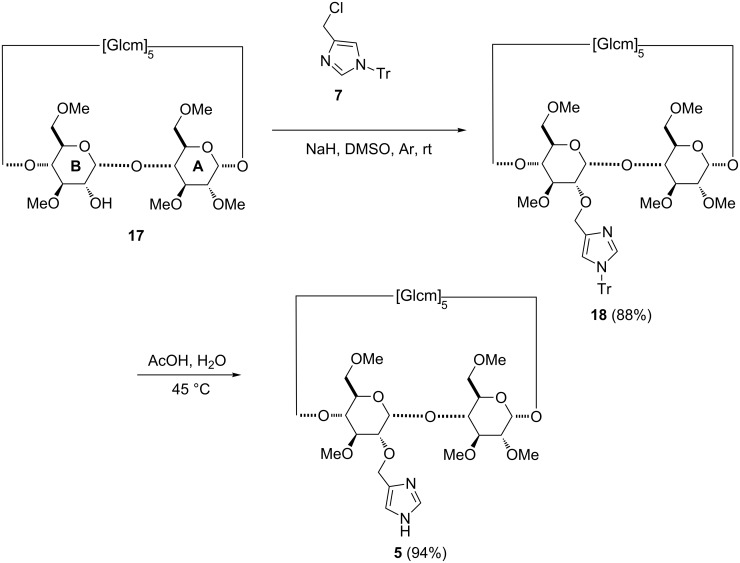
Synthesis of compound **5** (Tr = trityl).

The monosubstituted derivative **18** was prepared in good yield by reaction of the tritylated imidazole **7** with monohydroxy compound **17** in the presence of sodium hydride (Scheme 3) [35]. The ^1^H NMR signals of the diastereotopic methylene protons in product **18** were respectively observed at 4.57 and 4.71 ppm, while the corresponding ^13^C signal connected to the aromatic group was detected at 67.0 ppm. After deprotection under acidic conditions, the desired compound **5** was obtained in 94% yield.

### OPasic assays

A series of tests was undertaken with the aim to: i) estimate whether the length and flexibility of the linker between the imidazole and iodosobenzoate groups and the cyclodextrin affects the OPasic activity, ii) compare the results obtained with the scavengers with those obtained with a mixture of CD and imidazole or iodosobenzoate, and iii) study the structural influence of the organophosphorus compounds on the hydrolysis process.

**Figure 5:**
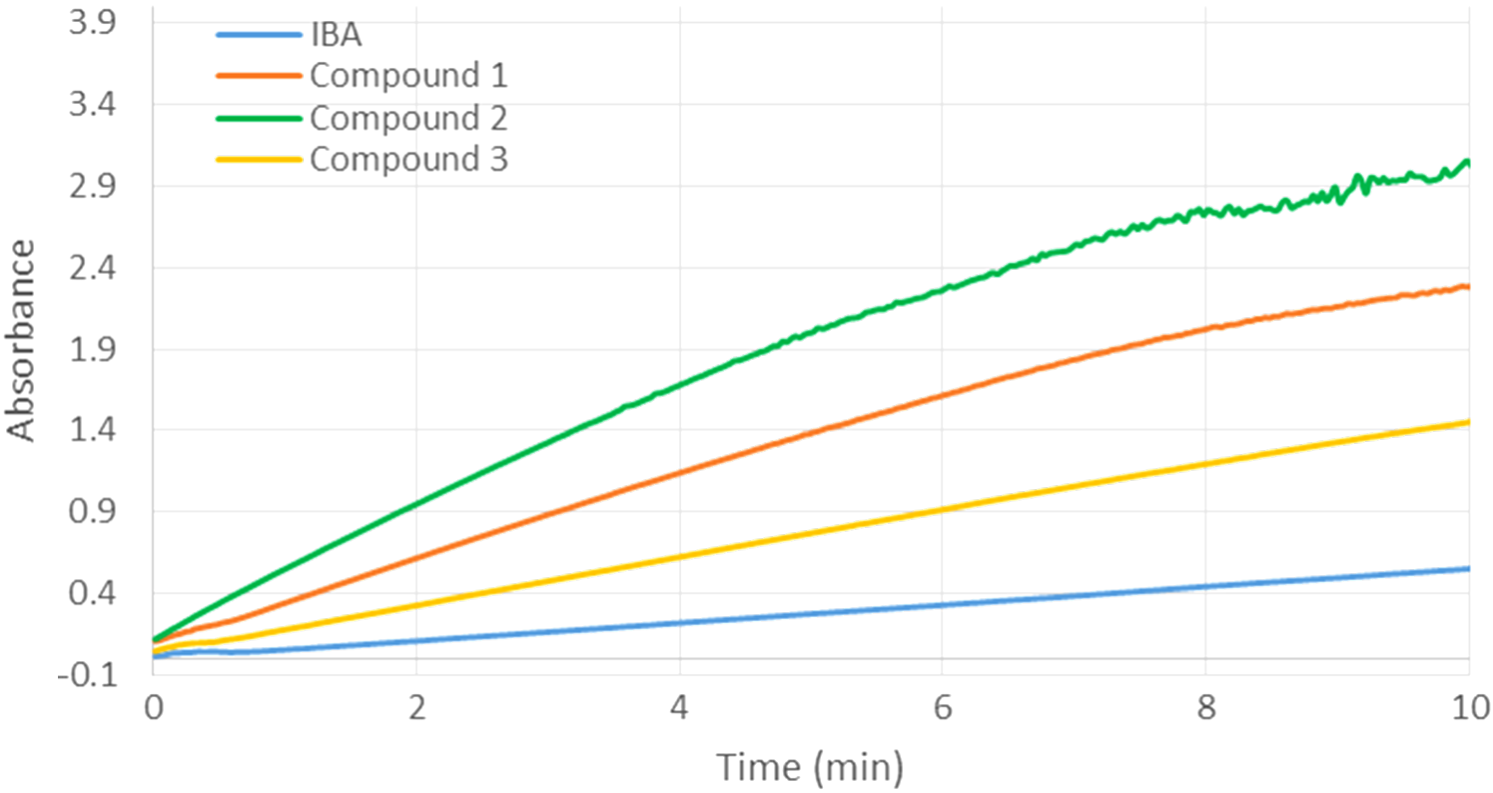
Hydrolysis of methyl paraoxon (0.5 mM) in the presence of compounds **1**, **2**, **3** or 2-iodosobenzoic acid (IBA) at 0.25 mM concentration.

In a first step, 2-iodosobenzoic acid (IBA) was used as reference compound to assess the efficiencies of derivatives **1**, **2**, and **3** towards the hydrolysis of methyl paraoxon (Figure 5). All scavengers **1**–**3** accelerated the transformation of the organophosphorus compound into *p*-nitrophenol compared to IBA alone. Compound **2** was the most effective, while the slowest pesticide hydrolysis was observed in the presence of derivative **3**. These results provided evidence that the relative position of the reactive group towards the phosphorus atom and the vicinity of the imidazole affect the efficiency of the scavengers. In fact, the pesticide degradation was more effective for the derivative possessing an *n*-propyl linker of the α-nucleophile to the macrocycle (**2** versus **1**). On the other hand the highest synergistic effect of the imidazole group was observed for scavengers connected with this group through a methylene, CH_2_ linker (**1** versus **3**).

**Figure 6:**
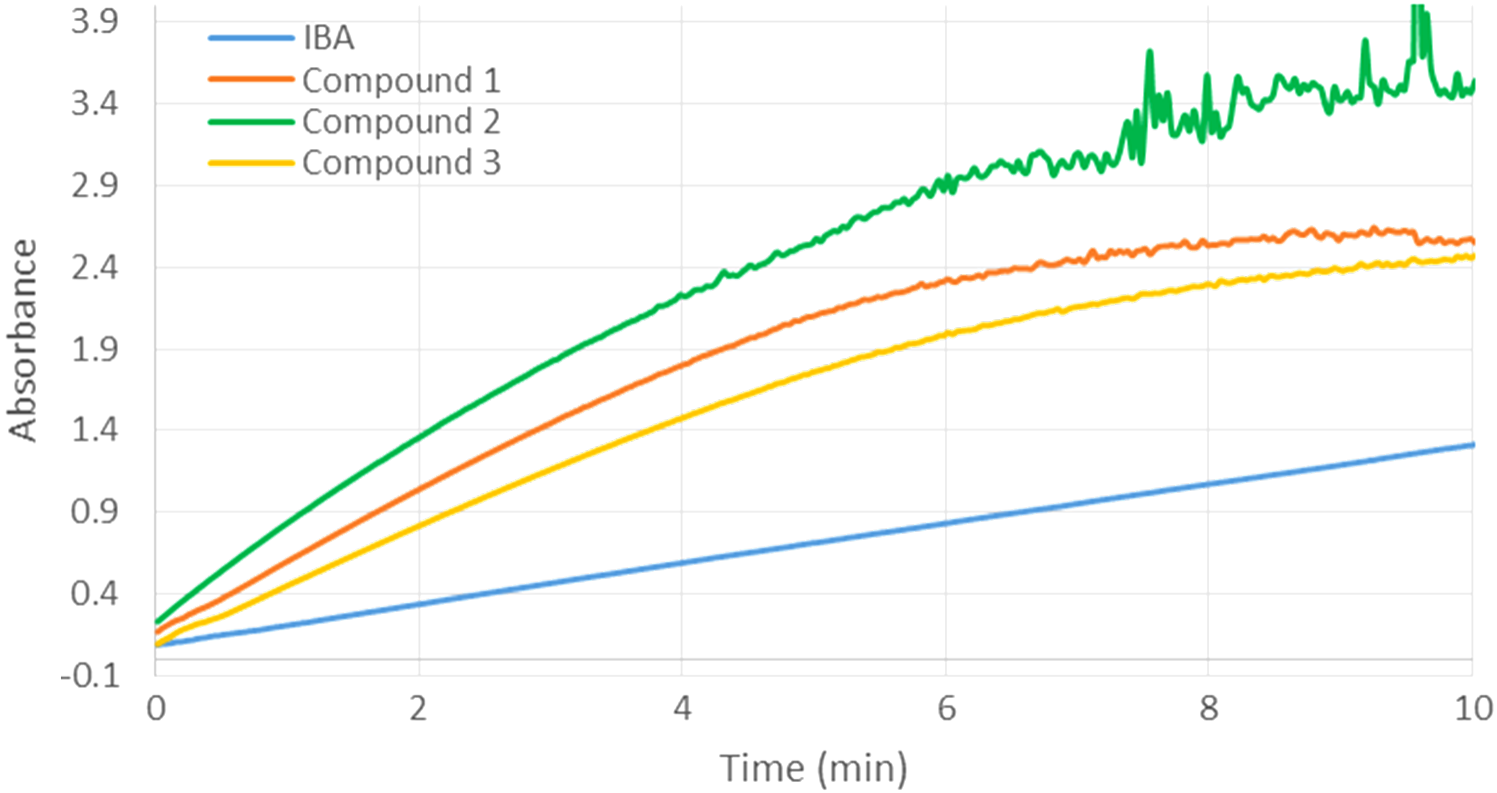
Hydrolysis of methyl paraoxon (0.5 mM) in the presence of compounds **1**, **2**, **3** or 2-iodosobenzoic acid (IBA) at 0.5 mM concentration.

At a higher concentration (0.5 mM) of scavengers **1**, **2** or **3**, we observed an improved efficiency for all three derivatives, compared to IBA (Figure 6) together with a reduced difference in their relative activities. For both studied concentrations (0.25 and 0.5 mM), the catalysts **1**–**3** were poisoned by the formed *p*-nitrophenol after a determined time. This phenomenon was already observed: the inclusion of the hydrolysis product in the cyclodextrin cavity limits the catalyst regeneration [27].

**Figure 7:**
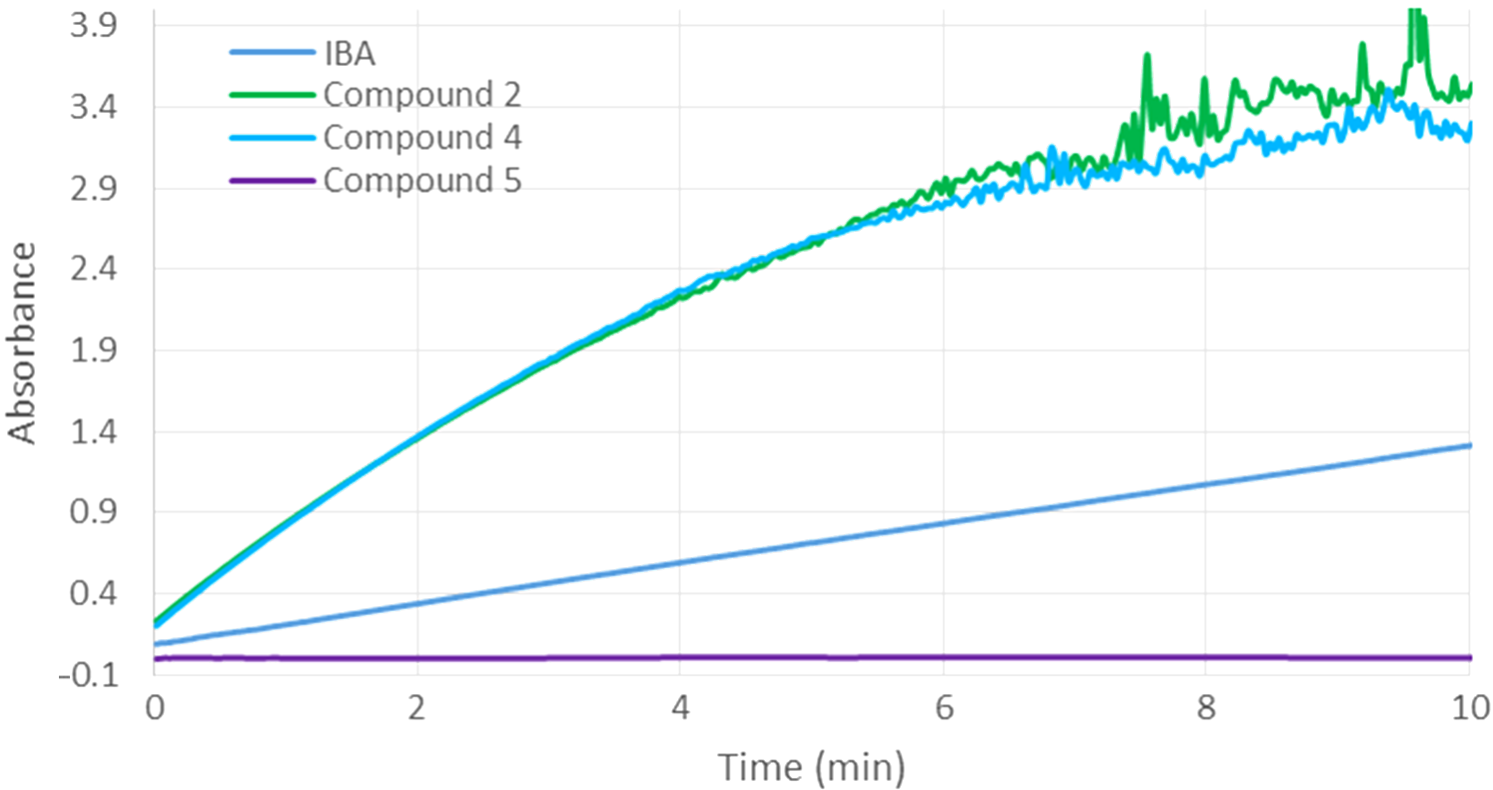
Hydrolysis of methyl paraoxon (0.5 mM) in the presence of compounds **2**, **4**, **5** or 2-iodosobenzoic acid (IBA) at 0.5 mM concentration.

Additional investigations were then performed with the monosubstituted cyclodextrin derivatives **4** and **5** (Figure 7). In the case of compound **5**, no activity was observed. In contrast, scavenger **4**, bearing only the α-nucleophile through a three-carbon atom linker showed a similar efficiency than **2**. Therefore, it can be concluded for compound **2** that there is no synergistic effect between the imidazole and the reactive groups. Thus, the imidazole substituent is able to accelerate the degradation of methyl paraoxon, induced by the covalently attached α-nucleophile iodosobenzoate, only if IBA is bound close to the macrocycle (**1** versus **3**).

**Figure 8:**
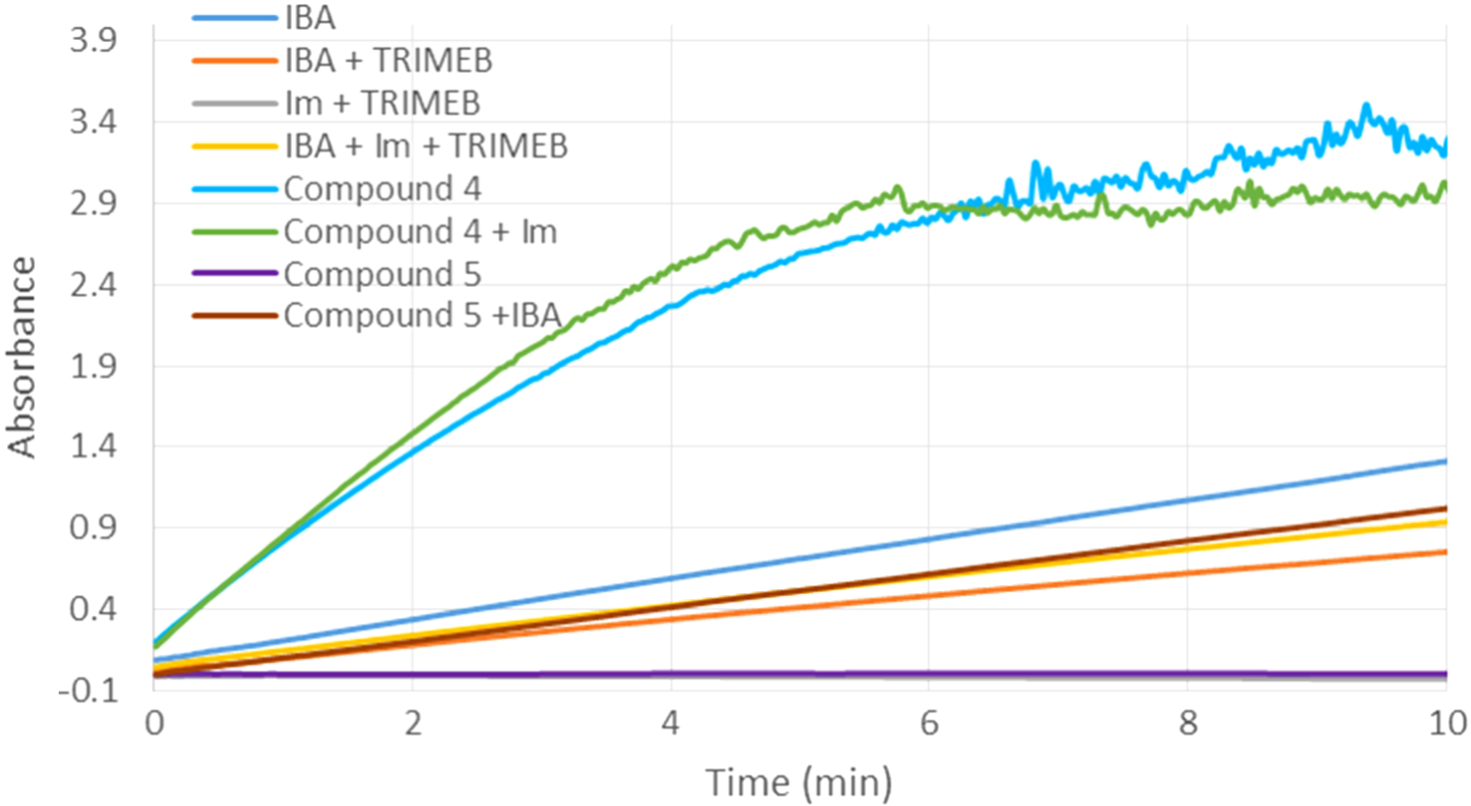
Hydrolysis of methyl paraoxon (0.5 mM) in the presence of mixtures of compounds **4**, **5** with IBA or imidazole and in the presence of a mixture of TRIMEB, 2-iodosobenzoic acid (IBA) and imidazole. The final concentrations of compounds **4**, **5**, 2-iodosobenzoic acid (IBA), imidazole and TRIMEB were 0.5 mM.

In the presence of both, imidazole and compound **4** (Figure 8), the rate of methyl paraoxon degradation was marginally increased with respect to scavenger **4** alone. On the other hand, a mixture of IBA and compound **5** (Figure 8) led to a reduced hydrolysis efficiency of IBA. The same effect was observed for a mixture of IBA, imidazole and TRIMEB. Furthermore, adding one molar equivalent of IBA to TRIMEB even strengthened the effect and it can be concluded that both, TRIMEB and compound **5** at least partially inhibited the pesticide hydrolysis by IBA. Therefore, when the α-nucleophile is not covalently bound to the macrocycle, the inclusion of paraoxon into the cyclodextrin cavity obviously protects the phosphate moiety of the pesticide from the external attack of the α-nucleophile. Clearly, the covalent binding of the α-nucleophile to the β-cyclodextrin scaffold leads to an increased hydrolytic activity.

The relative abilities of IBA and compounds **1**–**4** (0.25 mM) to accelerate the methyl paraoxon (0.5 mM) hydrolysis were then measured by determining the pseudo-first-order rate constants during the initial course of the process (Table 1). Under the conditions, the cyclodextrin derivatives **1**–**4** are fully active over the first 4 minutes. The absorbance increases linearly, thus indicating that no competitive inclusion of *p*-nitrophenol occurs as a side reaction and the catalyst is prevented from poisoning. In the presence of the most active compound **2**, 19.6% of paraoxon were hydrolyzed after 4 minutes whereas a conversion of only 2.6% was observed using IBA alone. The comparison with the reaction conducted in the presence of IBA revealed that scavengers **1**, **2**, **3** and **4** increased the catalytic factor by 5.5, 8.4, 2.9 and 8.2 times, respectively.

**Table 1:** Estimated pseudo-first-order rate constant (*k*) and amount (%) of hydrolyzed methyl paraoxon over the first 4 minutes.^a^

	IBA	**1**	**2**	**3**	**4**

*k* (10^−3^ min^−1^)	6.48	35.81	54.64	19.07	53.00
% of hydrolyzed methyl paraoxon	2.6	13.3	19.6	7.3	19.1

^a^Conditions: 20 mM phosphate buffer, 13 mM CTAC, 2.9 vol % DMSO, 3 vol % CH_3_OH at 25 °C. The concentrations of 2-iodosobenzoic acid (IBA), compounds **1**, **2**, **3** and **4** were 0.25 mM. The concentration of methyl paraoxon was 0.5 mM. The reactions were followed by UV–vis determination of the released *p*-nitrophenol at 400 nm.

**Figure 9:**
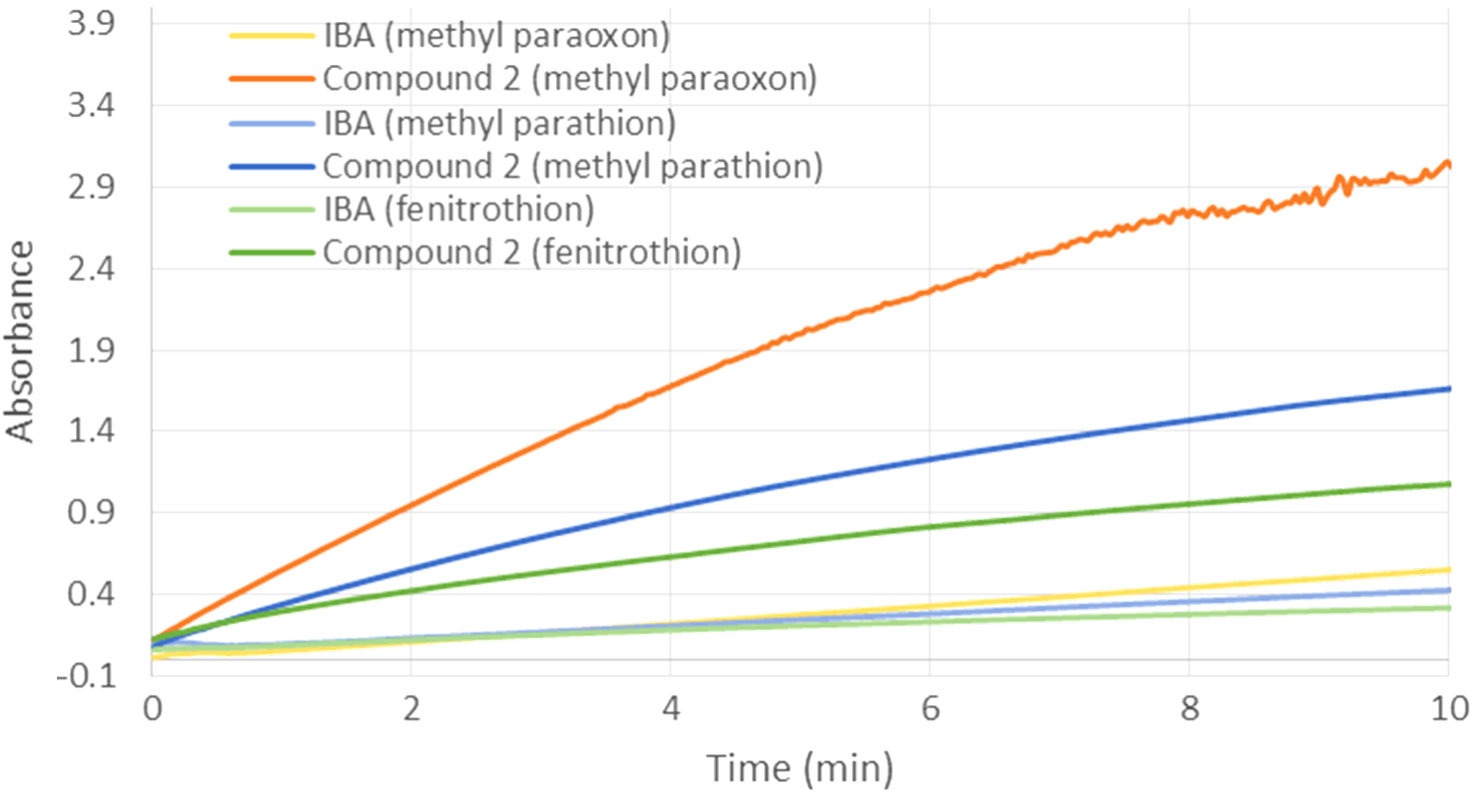
Influence of the pesticide structure on the hydrolytic efficiency of compound **2** (0.25 mM). Kinetic assays were carried out with methyl paraoxon, methyl parathion or fenitrothion (0.5 mM).

Derivative **2** appeared as the most promising disubstituted cyclodextrin for the degradation of methyl paraoxon. Therefore, this compound was subsequently used in the hydrolysis of other pesticides (Figure 9). Compound **2** was found to hydrolyze the organophosphorus compounds in the following order: fenitrothion < methyl parathion < methyl paraoxon while IBA alone displayed a generally lower and similar efficiency towards these three pesticides. This suggests that the electrophilic character of the phosphorus atom plays a role in the detoxification process (methyl parathion versus methyl paraoxon). Moreover, the strength and depth of the pesticide inclusion into the cyclodextrin cavity undeniably influence the degradation process. Indeed, it was shown that the lowest hydrolysis rates correspond to the highest dissociation constants from TRIMEB [36–37]. As methyl paraoxon represents the main and first metabolite of methyl parathion [38], scavenger **2** may be used in the late intervention after parathion intoxication.

**Figure 10:**
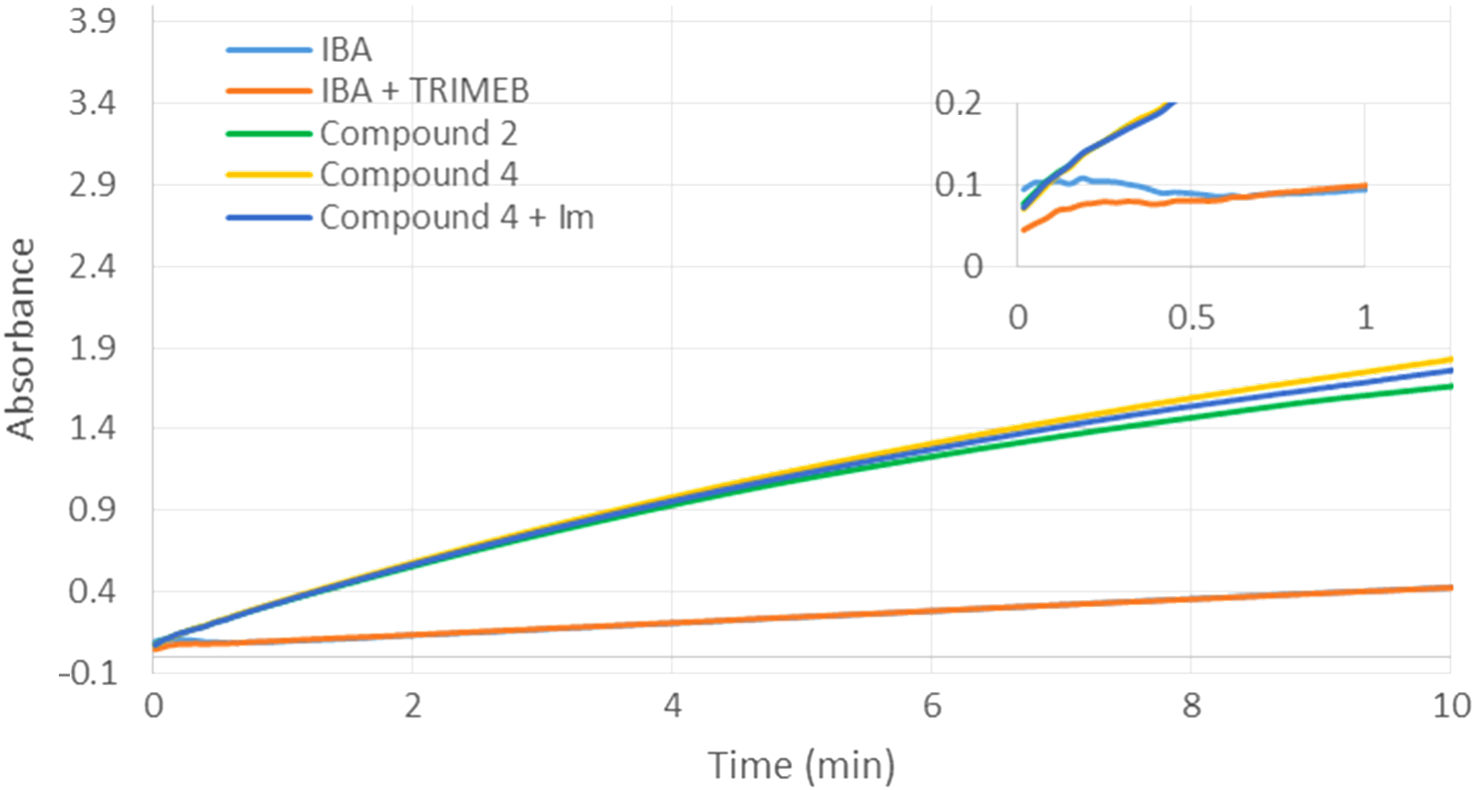
Influence of TRIMEB, IBA and imidazole on the hydrolysis of methyl parathion (0.5 mM). The final concentrations of compounds **2**, **4**, 2-iodosobenzoic acid (IBA), imidazole and TRIMEB were 0.25 mM.

The degradation of methyl parathion was also studied in presence of compound **4**. As already observed for methyl paraoxon compound **4** promoted the hydrolysis of methyl parathion with comparable efficiency than **2** (Figure 10). This suggests that the introduction of an imidazole group on the scavenger having the iodosobenzoate bound through an *n*-propyl linker has no effect on the hydrolytic activity, irrespective of the nature of the pesticide. Moreover, the hydrolysis rate of methyl parathion in the presence of imidazole and derivative **4** did not change when compared to scavenger **2**. This result confirms the importance to introduce the imidazole at a specific distance from the reactive group. It is important to note that, contrary to methyl paraoxon, in the case of methyl parathion, the use of an equimolar mixture of IBA and TRIMEB didn’t affect the degradation of the pesticide compared to IBA alone. The influence of the intermolecular interactions between the pesticides and the methylated oligosaccharide is of fundamental importance towards the degradation process.

The degradation efficiency of IBA and compounds **1** and **4** towards methyl parathion was also studied in terms of rate constants (Table 2) within the initial 4 minutes. In the presence of derivatives **1** and **4**, 10–11% hydrolysis of the substrate were observed and the catalytic factor increased by 7.1 and 7.5 times, respectively, compared to free IBA.

**Table 2:** Estimated pseudo-first-order rate constant (*k*) and amount (%) of hydrolyzed methyl parathion over the first 4 minutes.^a^

	IBA	**1**	**4**

*k* (10^−3^ min^−1^)	3.99	28.17	29.79
% of hydrolyzed methyl parathion	1.6	10.7	11.2

^a^Conditions: 20 mM phosphate buffer, 13 mM CTAC, 2.9 vol % DMSO, 3 vol % CH_3_OH at 25 °C. The concentrations of 2-iodosobenzoic acid (IBA), compounds **1** and **4** were 0.25 mM, respectively. The concentration of methyl parathion was 0.5 mM. The reactions were followed by UV–vis determination of the released *p*-nitrophenol at 400 nm.

**Figure 11:**
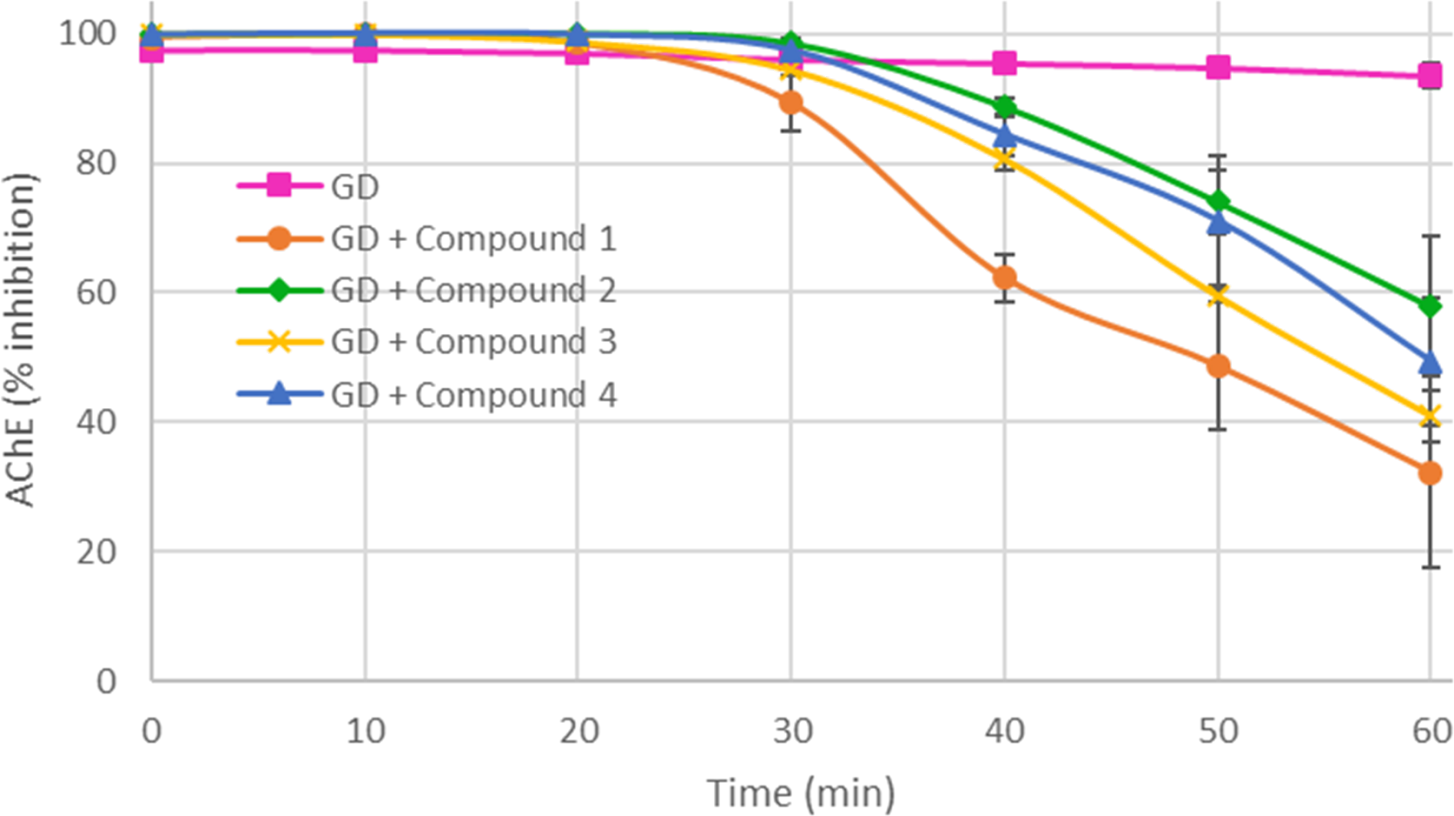
Ability of compounds **1**–**4** in preventing the inhibition of acetylcholinesterase by soman (GD).

Finally, the scavengers **1**–**4** were tested for their ability to prevent the inhibition of acetylcholinesterase by the chemical warfare agent soman (Figure 11). The heterodifunctionalized derivatives **1** and **3** were, this time, the most effective compounds. Preincubation of compounds **1** and **3** with soman for 60 minutes reduced the inhibition of the enzyme by 70% and 60%, respectively, whilst 40% and 50% reduction was observed with compounds **2** and **4** after the same pretreatment time. Moreover, scavengers **1** and **3** exhibited a detectable activity only after approximately 20 minutes incubation with soman, whereas compounds **2** and **4** showed an even later effect reducing the inhibition by 30% after 50 minutes pretreatment. Comparing the efficiencies of compounds **1** and **2** with those obtained for methyl paraoxon, we observed a higher activity when the α-nucleophile is close to the macrocycle. Concerning compounds **1** and **3**, the vicinity of the imidazole substituent to the active group confirmed its benefit to accelerate the soman degradation [33]. However, many parameters are involved in the detoxification process. It is known that an aromatic group attached to a phosphorus atom can interact with the cyclodextrin cavity wherefore soman and organophosphorus pesticides having an aromatic group were not hydrolyzed with the same efficiency by the scavengers. In fact, this efficiency is highly substrate-dependent and it is difficult to strictly correlate the hydrolysis profiles to the cyclodextrin structures of the prepared derivatives. Therefore, the design of new scavengers will require further structural studies on the inclusion complexes and affinity measurements with carefully selected analogs of organophosphates.

## Conclusion

New β-cyclodextrin derivatives were synthesized and their efficiency to degrade the organophosphorus pesticides and soman was evaluated. Some structural features of the scavengers could be identified as the key elements in charge to accelerate the decontamination of organophosphorus compounds: (1) the degradation kinetics of organophosphorus constituents is dependent on their affinity for the cyclodextrin cavity; (2) the α-nucleophile needs to be covalently bound to the macrocycle in order to enhance its activity towards the hydrolysis of organophosphorus compounds; (3) the hydrolysis process of pesticides bearing a phenyl group is mainly affected by the chain length of the linker between the iodosobenzoate group and the methylated oligosaccharide; (4) in case of soman, the degradation is enhanced by a cooperative effect observed between the imidazole and 2-iodosobenzoate when the latter is in close proximity to the macrocycle. A more extended structure–activity relationship study is envisaged to further investigate and better rationalize all parameters involved in this complex hydrolytic process.

## Supporting Information

File 1 Experimental procedures, NMR spectra, hydrolytic and detoxification assays.
