# Supplementary material for: Structure–efficiency relationships of cyclodextrin scavengers in the hydrolytic degradation of organophosphorus compounds
Source: Beilstein J Org Chem. 2017 Mar 6;13:417–27. doi: 10.3762/bjoc.13.45 (PMC5355938; doi:10.3762/bjoc.13.45)
Supplement: File 1 — Experimental procedures, NMR spectra, hydrolytic and detoxification assays. [file Beilstein_J_Org_Chem-13-417-s001.pdf]

Supporting Information

for

Structure–efficiency relationships of cyclodextrin scavengers in the hydrolytic degradation of organophosphorus compounds

Sophie Letort[1], Michaël Bosco[1], Benedetta Cornelio[1], Frédérique Brégier[1], Sébastien Daulon[2], Géraldine Gouhier[1] and François Estour[1]*

Address: [1]Normandie Univ, INSA Rouen, UNIROUEN, CNRS, COBRA (UMR 6014), 76000 Rouen, France and [2]DGA Maîtrise NRBC, Département Evaluation des effets des agents chimiques, 91710 Vert le Petit, France

Email: François Estour* - francois.estour@univ-rouen.fr

*Corresponding author

Experimental procedures, NMR spectra, hydrolytic and detoxification assays

Contents

General S2

Experimental procedures and data analysis for the synthesis of compounds **2–5** S4

^{1}H NMR and ^{13}C NMR spectra of compounds **13**, **14**, **16**, and **18** S12

Hydrolytic assays of pesticides S17

Detoxification assays of organophosphorus agents S18

1. General

All syntheses were monitored by thin-layer chromatography (TLC) on silica gel plates 60 F254 (E. Merck, Darmstadt, Germany). Developed plates were visualized using ultraviolet light and/or by dipping into a solution of sulfuric acid in ethanol (8%) followed by heating with a heat gun. Column chromatography was performed using silica gel 60 (0.04–0.063 mm, E. Merck) or 90 C18 (Zeoprep). Flash chromatography was performed on an automated apparatus Reveleris® (Grace Davison Discovery Sciences).

The compounds 4-chloromethyl-1-(triphenylmethyl)-imidazole (**7**) [1,2], 4-(3-chloropropyl)-1-(triphenylmethyl)imidazole (**8**) [1,3], methyl 5-(bromomethyl)-2-iodobenzoate (**11**) [4] and 5-(3-bromopropyl)-2-iodobenzoate (**12**) [5] used for the substitution of cyclodextrin derivatives were prepared as previously described. $2^A,3^B,6^A,6^B$-Tetra-*O*-methyl-pentakis-(2,3,6-tri-*O*-methyl)cyclomaltoheptaose (**6**) [6], 2^B-*O*-({1-trityl-1*H*-imidazol-4-yl}methyl)-$2^A,3^B,6^A,6^B$-tetra-*O*-methyl-pentakis-(2,3,6-tri-*O*-methyl)cyclomaltoheptaose (**9**) [7], 2,6-di-*O*-methyl-hexakis-(2,3,6-tri-*O*-methyl)cyclomaltoheptaose (**15**) [6], and 3,6-di-*O*-methyl-hexakis-(2,3,6-tri-*O*-methyl)cyclomaltoheptaose (**17**) [8] used as the starting materials were prepared as previously described.

Melting points were determined on a Kofler hot plate melting point apparatus and are uncorrected. UV spectra were recorded on a Varian's CARY© 50 UV–vis spectrophotometer. IR spectra were obtained on a Shimadzu IR 408 spectrometer and absorbance bands are expressed in cm^{-1} with only noteworthy absorptions listed. Routine NMR ^1H and ^{13}C NMR spectra were obtained in case of β-cyclodextrin derivatives **2**, **3**, **4**, **5**, **10**, **13**, **14**, **16** and **18**. The NMR spectra were recorded on a Bruker Avance 300 instrument spectrometer, chemical shifts (δ) are given in ppm and coupling constants (*J*) are given in hertz. All NMR experiments were performed at 300 MHz (^1H), and 75 MHz (^{13}C) in $CDCl_3$ at 300 K. The ^{13}C signals are given with one decimal place except when the values are too close (two decimal places). HRMS and MS analyses were performed on a QTOF Synapt G2-Si (Waters, Manchester, UK), equipped with an electrospray ionization (ESI) source (Z-spray) and an additional sprayer for the reference compound (Lock spray).

The Ellman solution needed for the detoxification assays of soman by compounds **1**, **2**, **3**, or **4** was prepared by dissolving acetylthiocholine (151 mg), 5,5'-dithiobis(2-nitrobenzoic acid) (100 mg), and

sodium bicarbonate (50 mg) in 25 mL of phosphate buffer (pH 7). The so-obtained solution was ten-fold diluted. Acetylcholinesterase (AChE) from human erythrocytes was purchased from Sigma Aldrich and the residual AChE activity was determined on a microplate reader PowerWave XS(R) (Biotek).

References

[1] Graßmann S.; Apelt J.; Sippl W.; Ligneau X.; Pertz H.H.; Zhao Y.H.; Arrang J.-M.; Robin Ganellin C.; Schwartz J.-C.; Schunack W.; Starck H., *Bioorg. & Med. Chem.*, **2003**, *11*, 2163-2174.

[2] Houssin R.; Pommery J.; Salaün M.C.; Deweer S.; Goossens J.F., *J. Med. Chem.*, **2002**, *45*, 533-536.

[3] Ganellin C.R.; Fkyerat A.; Bang-Andersen B.; Athmani S.; Tertiuk W; Garbarg M.; Ligneau X.; Schwartz J.C., *J. Med. Chem.*, **1996**, *39*, 3806-3813.

[4] Masurier N.; Estour F.; Froment M.-T.; Lefèvre B.; Debouzy J.-C.; Brasme B.; Masson P.; Lafont O., *Eur. J. Med. Chem.*, **2005**, *40*, 615-623.

[5] Kalakuntla R.K.; Wille T.; Le Provost R.; Letort S.; Reiter G.; Müller S.; Thiermann H.; Worek F.; Gouhier G.; Lafont O.; Estour F., *Toxicol. Lett.*, **2013**, *216*, 200-205.

[6] Xiao, S.; Yang, M.; Sinaÿ, P.; Blériot, Y.; Sollogoub, M.; Zhang, Y., *Eur. J. Org. Chem.*, **2010**, 1510–1516.

[7] Letort, S.; Mathiron, D.; Grel, T.; Albaret, C.; Daulon, S.; Djedaini-Pilard, F.; Gouhier, G.; Estour F., *Chem. Commun.* **2015**, *51*, 2601-2604.

[8] Cousin, H.; Cardinael, P.; Oulyadi, H.; Pannecoucke, X.; Combret, J.-C., *Tetrahedron: Asymmetry*, **2001**, *12*, 81–88.

2. Experimental procedures for the synthesis of compounds 2–5

2.1 Synthesis of disubstituted compounds 2 and 3

2.1.1 Synthesis of compound 2

2^B-O-({1-Trityl-1H-imidazol-4-yl}methyl)-3^A-O-(3-carboxypropyl-4-iodobenzyl)-2^A,3^B,6^A,6^B-tetra-O-methyl-pentakis-(2,3,6-tri-O-methyl)cyclomaltoheptaose (13): NaH (60% in mineral oil, 0.028 g, 0.697 mmol) was added to a solution of compound **9** (0.400 g, 0.232 mmol) in 3 mL of anhydrous dimethyl sulfoxide at room temperature under argon. The reaction mixture was stirred at room temperature for 7 hours. Methyl 5-(3-bromopropyl)-2-iodobenzoate (0.267g, 0.697 mmol) in 0.9 mL of dimethyl sulfoxide was added and the reaction mixture stirred at room temperature overnight. Water (5 mL) was added dropwise to quench the reaction and the reaction mixture was extracted with ethyl acetate (3 × 30 mL). The organic layers were collected, washed with 1 N aqueous solution of HCl (2 × 10 mL), then brine (10 mL), and dried over magnesium sulfate. The solvent was removed under reduced pressure. The residue was purified by silica gel chromatography (dichloromethane/ethyl acetate/methanol 5/5/0.5, v/v/v) to give the desired product as a white powder (174 mg, yield 37%. **^{1}H-NMR (300 MHz, $CDCl_3$)**: δ 1.66-1.86 (m, 2H, $OCH_2C\underline{H_2}CH_2$-Ph), 2.60 (t, J= 7.5 Hz, 2H, $OCH_2CH_2C\underline{H_2}$-Ph), 3.11 (dd, ^{3}J= 9.0 Hz, ^{3}J= 3.0 Hz, 1H, 1 x H-2), 3.17-3.22 (m, 5H, 5 x H-2), 3.32 (s, 3H, 1 x $OCH_{3(6)}$), 3.39 (m, 19H, 6 x $OCH_{3(6)}$, 1 x H-2), 3.46-3.67 (m, 57H, 6 x $OCH_{3(2)}$, 6 x $OCH_{3(3)}$, 7x H-3, 7 x H-4, 7x H-6), 3.69-4.00 (m, 16H, 7 x H- 5, 7x H'-6, $O\underline{CH_2}CH_2CH_2$-Ph), 3.89 (s, 3H, OCH_3), 4.58-4.74 (ABq spectrum, ^{2}J= 12.7 Hz, 2H, CH_2-Im), 5.03 (d, ^{3}J= 3.0 Hz, 1H, 1 x H-1), 5.10-5.19 (m, 6H, 6 x H-1), 6.89 (s, 1H, H-Im), 6.95 (dd, ^{3}J= 9.0Hz, ^{4}J= 3.0Hz, 1H, H-Ph), 7.09-7.12 (m, 6H, H- Ph_{Tr}), 7.29-7.35 (m, 9H, H-Ph_{Tr}), 7.37 (s, 1H, H-Im), 7.59 (d, ^{4}J= 3.0 Hz, 1H, H-Ph), 7.77 (d, ^{3}J= 9.0 Hz, 1H, H-Ph). **^{13}C-NMR (75 MHz, $CDCl_3$)**: δ 31.8, 31.9 ($OCH_2\underline{C}H_2CH_2$-Ph, $OCH_2CH_2\underline{C}H_2$-Ph), 52.5 ($COO\underline{C}H_3$), 58.4, 58.5, 58.6, 58.92, 58.96, 59.04, 59.10 [13C, 6 x OCH_3(C-2), 7 X OCH_3(C-6)], 61.28, 61.33, 61.4, 61.5, 61.73, 61.76 [6C, 6 x OCH_3(C-3)], 67.0 (OCH_2-Im), 70.77, 70.83, 70.87, 71.00, 71.06, 71.12, 71.3, 71.4, 71.6, 71.7, 71.8 (14C, 7 x C-5, 7 x C-6), 73.0 ($O\underline{C}H_2CH_2CH_2$-Ph), 75.4 ($C_{IV}$-Tr), 79.69, 79.71, 79.9, 80.0, 80.3, 80.4, 80.7, 80.8, 81.5, 81.79, 81.83, 81.9, 81.98, 82.02, 82.10, 82.12, 82.2, 82.3 (21C, 7 x C-2, 7 x C-3, 7 x C-4), 90.4 (C-I), 98.8, 98.9, 99.0, 99.1, 99.2 (7C, C-1), 120.4 (CH-Im), 128.1, 128.2, 129.8 (CH-Ph_{Tr}), 131.3 (CH-Ph), 133.3 (CH-Ph), 134.8 (C-Ph), 138.7, 138.8 (2C, C-Ph, C-Im), 141.0 (CH-Im), 142.5 (3C, C-Ph_{Tr}), 143.2 (CH-Ph), 167.1 (CO). **ESI-MS (m/z)**: 2025 $[M+H]^+$. **Elemental analysis**: Anal. Calcd for $C_{95}H_{137}IN_2O_{37}$: C, 56.32; H, 6.82; N, 1.38. Found: C, 55.61; H, 6.79; N, 1.39. **HRMS** calcd

for $C_{95}H_{138}N_2O_{37}I$: 2025.8023, found: 2025.8013. **HRMS** calcd for $C_{95}H_{137}N_2O_{37}NaI$: 2047.7843, found: 2047.7888. **IR** (KBr): 3431, 2931, 1658 cm^{-1}. **Melting Point**: >260°C.

2^B-O-({1H-Imidazol-4-yl}methyl)-3^A-O-(3-{4-carboxy-3-iodosophenyl}propyl)-2^A,3^B,6^A,6^B-tetra-O-methyl-pentakis-(2,3,6-tri-O-methyl)cyclomaltoheptaose (2): Heterodifunctionalized compound **13** (0.082 g, 0.04 mmol) was dissolved in a mixture of acetic acid (1 mL) and water (1.24 mL). Then, sodium periodate (10.845 g, 4.0 mmol) was added and the mixture stirred for 24 hours at 45 °C. After cooling to room temperature, water (4.12 mL) was added to solubilize the excess of sodium periodate. The solution was extracted with dichloromethane (3 × 16 mL) and the combined organic layers were dried over magnesium sulfate. After filtration, the solvent was removed under reduced pressure. The crude product was purified by reversed phase chromatography (gradient: water (100%) to water/acetonitrile (70/30, v/v) to give the desired product as a white powder (0.53 g, yield 75%). **^{1}H-NMR (300 MHz, $CDCl_3$)**: δ 1.86-2.04 (m, 2H, $OCH_2C\underline{H_2}CH_2$-Ph), 2.65-2.96 (m, 2H, $OCH_2CH_2C\underline{H_2}$-Ph), 3.18-3.21 (m, 6H, 6 x H-2), 3.27-3.38 (m, 22H, 7 x $OCH_{3(6)}$, 1 x H-2), 3.50-4.01 (m, 73H, 6 x $OCH_{3(2)}$, 6 x $OCH_{3(3)}$, 7 x H-3, 7 x H-4, 7 x H- 5, 14 x H-6, $OC\underline{H_2}CH_2CH_2$-Ph), 4.36-4.54 (m, 2H, CH_2-Im), 4.96-5.30 (m, 7H, H-1), 6.98 (s, 1H, H-Im), 7.55-7.63 (m, 2H, H-Ph, H-Im), 7.82-7.84 (m, 1H, H-Ph), 7.96 (s, 1H, H-Ph). **^{13}C-NMR (75 MHz, $CDCl_3$)**: δ 31.6 ($OCH_2\underline{C}H_2CH_2$-Ph, $OCH_2CH_2\underline{C}H_2$-Ph), 58.3, 58.4, 58.5, 58.9, 59.0 [13C, 6 x OCH_3 (C-2), 7 x OCH_3 (C-6)], 61.2, 61.3, 61.4, 61.5, 61.7 [6C, 6 x OCH_3,(C-3)], 65.2 (OCH_2-Im), 70.7, 70.8, 71.0, 71.1, 71.4, 71.5,71.6, (14C, 7 x C-5, 7 x C-6), 73.6 (OCH_2-Ph), 79.6, 80.2, 80.4, 80.5, 80.8, 81.7, 81.8, 81.9, 82.0, 82.1, 82.2 [21C, 7 x C-2, 7 x C-3, 7 x C-4], 98.2, 98.8, 98.9, 99.1 (7C, C-1), 117.4 (2C, CH-Im, C-IO), 125.9 (CH-Ph), 131.0 (CH-Ph), 131.6 (C-Ph), 134.8 (C-Ph), 135.3 (CH-Im), 145.5 (CH-Ph),169.6 (CO). The signal corresponding to C-Im is not identified. **ESI-MS (m/z)**: 1819 $[M+Cl]^-$. **HRMS** Calcd for $C_{75}H_{121}N_2O_{38}ClI$: 1819.6331, Found: 1819.6395. **IR (KBr)**: 3440, 2928, 1656 cm^{-1}. **Melting Point**: >260°C.

2.1.2 Synthesis of compound 3

2^B-O-({1-Trityl-1H-imidazol-4-yl}propyl)-3^A-hydroxy-2^A,3^B,6^A,6^B-tetra-O-methyl-pentakis-(2,3,6-tri-O-methyl)cyclomaltoheptaose (10): NaH (60% in mineral oil, 0.047 g, 11.9 mmol) was added to a solution of compound **6** (1.67g, 1.19 mmol) in 6.7 mL of anhydrous dimethyl sulfoxide at room temperature under argon. The reaction mixture was stirred at room temperature for 7 hours. 4-(3-Chloropropyl)-1-trityl-imidazole (0.460 g, 1.19 mmol) in 0.9 mL of dimethyl sulfoxide was then added and the reaction mixture was stirred at room temperature overnight. Water was added dropwise to quench the reaction and the reaction mixture was

extracted with ethyl acetate (3 × 40 mL). The organic layers were collected, washed with brine (30 mL), and dried over magnesium sulfate. After filtration, the solvent was removed under reduced pressure and the residue was purified by chromatography (toluene/acetone, 45/55; v/v) to give the desired product as a white powder (0.72 g, yield 35%). **^{1}H-NMR (300 MHz, $CDCl_3$)**: δ 1.90-2.01 (m, 2H, $OCH_2C\underline{H_2}CH_2$-Im), 2.60 (t, ^{3}J= 9.0 Hz, 2H, $OCH_2CH_2C\underline{H_2}$-Im), 3.14-3.20 (m, 6H, 6 x H-2), 3.25 (dd, ^{3}J= 9.0 Hz, ^{3}J= 3.0 Hz, 1 x H-2), 3.39 (m, 21H, 7 x $OCH_{3(6)}$), 3.44-3.64 (m, 56H, 6 x $OCH_{3(2)}$, 6 x $OCH_{3(3)}$, 6 x H-3, 7 x H-4, 7 x H-6), 3.69-3.87 (m, 16H, 7 x H-5, 7 x H-6', $OC\underline{H_2}CH_2CH_2$-Im), 3.94 (t, ^{3}J= 9.0 Hz, 1H, H-3), 4.92 (d, J= 3.3 Hz, 1H, H-1), 5.04 (d, ^{3}J= 3.3 Hz, 1H, H-1), 5.09-5.12 (m, 5H, 5 x H-1), 6.57 (s, 1H, H-Im), 7.11-7.16 (m, 6H, H-Ph_{Tr}), 7.30-7.34 (m, 9 H, H-Ph_{Tr}), 7.37(s, 1H, H-Im). **^{13}C-NMR (75 MHz, $CDCl_3$)**: δ 24.5 (OCH_2-CH_2-$\underline{C}H_2$-Im), 29.2 (OCH_2-$\underline{C}H_2$-CH_2-Im), 58.35, 58.36, 58.42, 58,45, 58.51, 58.54, 58.90, 58.97 [13C, 6 x OCH_3(C-2), 7 x OCH_3(C-6)], 61.35, 61.37, 61.39, 61.5, 61.6, 61.8, [6C, 6 x OCH_3(C-3)], 70.0, 70.9, 71.0, 71.2, 71.30, 71.36 (14C, 7 x C-5, 7 x C-6), 71.7 (C-3), 72.3 ($O\underline{C}H_2CH_2CH_2$-Im), 75.1 ($C_{IV}$-trityl), 80.06, 80.09, 80.2, 80.7, 81.2, 81.43, 81.47, 81.51, 81.55, 81.62, 81.65, 81.70, 81.98, 82.01, 82.1, 82.2, 82.3, 83.2 (20C, 7 x C-2, 6 x C-3, 7 x C-4), 98.7, 98.8, 99.0, 99.3, 99.6, 99.8 (6C, 6 x C-1), 101.0 (C-1), 118.0 (CH-Im), 128.0, 129.7 (15C, CH-Ph_{Tr}), 138.2 (C-Im), 140.5 (CH-Im), 142.4 (3C, C-Ph_{Tr}). **ESI-MS (m/z)**: 1751 $[M+H]^+$. **Elemental analysis**: Anal. Calcd for $C_{86}H_{130}N_2O_{35}\cdot 5H_2O$: C, 56.08; H, 7.66; N, 1.52. Found: C, 58.26; H, 7.52; N, 1.39. **IR:** 2926 cm^{-1}. **Melting Point**: >260°C.

2^B-O-({1-Trityl-1H-imidazol-4-yl}propyl)-3^A-O-(3-carboxymethyl-4-iodobenzyl)-2^A,3^B,6^A,6^B-tetra-O-methyl-pentakis-(2,3,6-tri-O-methyl)cyclomaltoheptaose (14): NaH (60% in mineral oil, 0.027 g, 0.686 mmol) was added to a solution of compound (**10**, 0.400 g, 0.229 mmol) in 3 mL of anhydrous dimethyl sulfoxide at room temperature under argon. The reaction mixture was stirred at room temperature for 7 hours. Then, methyl 5-bromomethyl-2-iodobenzoate (0.243 g, 0.686 mmol) in 0.9 mL of anhydrous dimethyl sulfoxide was added and the reaction mixture was stirred at room temperature overnight. Water (5mL) was added dropwise to quench the reaction. The reaction mixture was extracted with ethyl acetate (3 × 30 mL). The organic layer was washed with 1 N aqueous solution of HCl (2 × 10 mL), brine (10 mL) and dried over magnesium sulfate. After filtration, the solvent was removed under reduced pressure and the residue was purified by silica gel chromatography using dichloromethane/ethyl acetate/methanol (5/5/0.5; v/v/v) to give the desired product as a white powder (125 mg, yield 27%). **^{1}H-NMR (300 MHz, $CDCl_3$)**: δ 1.65-1.69 (m, 2H, $OCH_2C\underline{H_2}CH_2$-Im), 2.41-2.58 (m, 2H, $OCH_2CH_2C\underline{H_2}$-Im), 3.09-3.24 (m, 7H, 7 x H-2), 3.29-3.42 (m, 22H, 7 x $OCH_{3(6)}$, H-3A), 3.44-3.92 (m, 72H, 6 x $OCH_{3(2)}$, 6 x $OCH_{3(3)}$, 7x H-4, 7 x H-5, 14 x H-6, 1H, $OC\underline{H_2}CH_2CH_2$-

Im), 3.82 (s, 3H, OCH_3), 4.01 (dd, J= 10.8, 2.6 Hz, 1H, $OC\underline{H_2}CH_2CH_2$-Im), 4.68 (d, J= 12.2 Hz, 1H, CH_2-Ph), 5.01 (d, ^{3}J= 3.2 Hz, 1H, H-1), 5.04-5.22 (m, 7H, 6 x H-1, CH_2-Ph), 6.48 (s, 1H, H-Im), 7.04-7.14 (m, 6H, H- Ph_{Tr}), 7.21-7.32 (m, 10H, H-Ph_{Tr}, H-Im), 7.34 (1H, H-Im), 7.78-7.87 (m, 2H, 2 x H-Ph). **^{13}C-NMR (75 MHz, $CDCl_3$)**: δ 24.5 ($OCH_2CH_2\underline{C}H_2$-Im), 29.5 ($OCH_2\underline{C}H_2CH_2$-Im), 52.3 ($COO\underline{C}H_3$), 58.32, 58.45, 58.52, 58.60, 58.98, 59.03, 59.05, 59.07 [13C, 6 x OCH_3(C-2), 7 x OCH_3(C-6)], 61.24, 61.26, 61.4, 61.5, 61.68, 61.71 [6C, 6 x OCH_3(C-3)], 70.3 (C-3A), 70.75, 70.79, 70.93, 70.96, 71.05, 71.12, 71.17, 71.28, 71.34, 71.68, (15C, 7 x C-5, 7 x C-6, $O\underline{C}H_2CH_2CH_2$-Im), 74.4 ($OCH_2$-Ph), 75.2 ($C_{IV}$-Tr), 79.6, 79.8, 80.1, 80.3, 80.4, 80.75, 80.79, 81.04, 81.47, 81.75, 81.79, 81.89, 81.90, 81.94, 81.99, 82.0, 82.1, 82.20, 82.23 (20C, 7 x C-2, 6 x C-3, 7 x C-4), 92.1 (C-I), 98.5, 98.98, 99.05, 99.13, (7C, C-1), 118.1 (CH-Im), 128.0 (3C, CH-Ph_{Tr}), 128.01 (6C, CH-Ph_{Tr}), 129.8 (6C, CH-Ph_{Tr}), 130.2 (CH-Ph), 132.01 (C-Ph), 134.5 (C-Ph), 138.3 (CH-Im), 140.3 (C-Ph), 140.8 (C-Im), 141.0 (CH-Ph), 142.5 (3C, C-Ph_{Tr}**)**, 167.0 (CO). **ESI-MS (m/z)**: 2025 $[M+H]^+$. **HRMS** Calcd for $C_{95}H_{138}IN_2O_{37}$: 2025.8023, Found: 2025.8009. **IR** (KBr): 3439, 2935, 1632 cm^{-1}. **Melting Point**: >260°C.

2^B-O-(3-{4-Carboxy-3-iodosophenyl}propyl)-3^A-O-({1H-imidazol-4-yl}methyl)-2^A,3^B,6^A,6^B-tetra-O-methyl-pentakis-(2,3,6-tri-O-methyl)cyclomaltoheptaose (3): Heterodifunctionalized compound **14** (0.1g, 0.049 mmol) was solubilized in a solution of acetic acid (30% in water, v/v, 1.14 mL or 1.12 mL). Then, sodium periodate (1.06 g, 4.9 mmol) was added and the mixture stirred for 24 hours at 45 °C. After cooling to room temperature, the mixture was filtered to remove the excess of sodium periodate. The filtrate was extracted with dichloromethane (3 × 30 mL) and diethyl ether (2 × 20 mL). The combined organic layers were dried over magnesium sulfate. After filtration, the volatiles were removed under reduced pressure. The crude product was purified by reversed phase chromatography (gradient: water (100%) to water/acetonitrile (70/30, v/v) to give the desired product as a white powder (0.026 g, yield 30%). **^{1}H-NMR (300 MHz, $CDCl_3$):** δ 1.44-1.63 (m, 2H, $OCH_2C\underline{H_2}CH_2$-Im), 2.32-2.55 (m, 2H, $OCH_2CH_2C\underline{H_2}$-Im), 3.08-3.23 (m, 6H, 6 x H-2), 3.24-3.42 (m, 22H, 7 x $OCH_{3(6)}$, 1 x H-2), 3.43-4.26 (m, 73H, 6 x $OCH_{3(2)}$, 6 x $OCH_{3(3)}$, 7 x H-3, 7 x H-4, 7 x H- 5, 14 x H-6, $OC\underline{H_2}CH_2CH_2$-Im), 4.92-5.66 (m, 9H, 7 x H-1, CH_2Ph), 6.52 (s, 1H, H-Im), 7.52 (s, 1H, H-Im), 7.87 (d, J= 8.0 Hz, 1H, CH-Ph), 8.03 (d, J= 8.0 Hz, 1H, CH-Ph), 8.21 (s, 1H, CH-Ph). **^{13}C RMN (75 MHz, $CDCl_3$)**: δ 22.4 ($OCH_2CH_2\underline{C}H_2$-Im), 29.2 ($OCH_2\underline{C}H_2CH_2$-Im), 58.4, 58.58, 58.64, 58.7, 59.07, 59.10, 59.12, 59.21, 59.23, 59.3 [13C, 6 x OCH_3(C-2), 7 x OCH_3(C-6)], 61.2, 61.3, 61.5, 61.79, 61.82 [6C, 6 x OCH_3(C-3)], 69.8 (C-3A), 70.8, 71.10, 71.13, 71.15, 71.3, 71.36, 71.39, 71.5, 71.6, 71.7, 71.9 (15C, 7 x C-5,7 x C-6, $O\underline{C}H_2CH_2CH_2$-Im), 74.4

(OCH_2-Ph), 79.2, 79.5, 80.2, 80.35, 80.43, 80.6, 80.71, 80.74, 80.97, 81.2, 81.80, 81.81, 81.84, 81.98, 82.03, 82.24, 82.29, 82.33 (20C, 7 x C-2, 6 x C-3, 7 x C-4), 98.4, 98.93, 98.98, 99.0, 99.1, 99.2 (7C, 7 x C-1), 118.5 (2C, C-I, CH-Im), 125.9 (CH-Ph), 130.4 (CH-Ph), 130.9 (C-Ph), 131.0 (CH-Im), 133.8 (CH-Ph), 141.1 (C-Im), 143.3 (C-Ph), 172.7 (CO). **ESI-MS (m/z)**: 1785.7 $[M+H]^+$. **HMRS** calcd for $C_{75}H_{122}IN_2O_{38}$: 1785.6720, found: 1785.6732. **IR(KBr)**: 3436, 2929, 1646 cm^{-1}. **Melting Point**: >260°C.

2.2 Synthesis of monosubstituted compounds 4 and 5

2.2.1 Synthesis of compound 4

2,6-Di-O-methyl-3-O-({3-carboxy-4-iodophenyl}-propyl)-hexakis-(2,3,6-tri-O-methyl) cyclomaltoheptaose (16): NaH (60% in mineral oil) (78 mg, 1.95 mmol) was added to a solution of 2,6-di-O-methylhexakis-(2,3,6-tri-O-methyl)-cyclomaltoheptaose 15 (460 mg, 325 µmol) in 3 mL of anhydrous dimethyl sulfoxide at room temperature under argon atmosphere. The reaction mixture was stirred at room temperature for 1 hour. Then to the reaction mixture was added a solution of methyl 5-(3-bromopropyl)-2-iodobenzoate **12** (374 mg, 975 µmol) in anhydrous dimethyl sulfoxide (0.7 mL). The reaction mixture was stirred at room temperature for 14 hours. Water (20 mL) was added dropwise to quench the reaction. The reaction mixture was extracted with ethyl acetate (3 × 20 mL). The resulting aqueous layer containing the crude compound 16 was diluted with 60 mL of an aqueous solution of ammonium bicarbonate (0.25 mM). This solution was purified by column chromatography on reverse phase silica gel C18 (ammonium bicarbonate 0.25 M/acetonitrile, 100/0 to 65/35; v/v) to give the desired product (48 mg, 28 µmol) after freeze drying as a white foam. Yield = 15%. **^{1}H NMR (300 MHz, $CDCl_3$)**: δ 1.99 (q, ^3J = 7.3 Hz, 2H, $OCH_2C\underline{H_2}CH_2$-Ph), 2.52 – 2.78 (m, 2H, $OCH_2CH_2C\underline{H_2}$-Ph), 3.06 – 3.29 (m, 7H, 7 x H-2), 3.30 – 3.40 (m, 22H, 1 x H-3, 7 x $OCH_{3(6)}$), 3.41 – 4.10 (m, 75H, 14 x H-6, 7 x H-5, 7 x H-4, 6 x H-3, 6 x $OCH_{3(3)}$, 7 x $OCH_{3(2)}$, $OC\underline{H_2}CH_2CH_2$-Ph), 5.05 (d, ^3J = 3.2 Hz, 1H, 1 x H-1), 5.07 – 5.15 (m, 4H, 4 x H-1), 5.18 (d, ^3J = 3.4 Hz, 1H, 1 x H-1), 5.25 (d, ^3J = 3.5 Hz, 1H, 1 x H-1), 7.00 (dd, ^3J = 8.0 Hz, ^4J = 1.8 Hz, 1H, H-Ph), 7.66 (d, ^4J = 1.8 Hz, 1H, H-Ph), 7.86 (d, ^3J = 8.0 Hz, 1H, H-Ph). **^{13}C NMR (75 MHz, $CDCl_3$)**: δ 31.4 ($OCH_2\underline{C}H_2CH_2$-Ph), 31.9 ($OCH_2CH_2\underline{C}H_2$-Ph), 58.5, 58.56, 58.61, 59.05, 59.09, 59.2 [14C, 7 x OCH_3(C-2), 7 x OCH_3(C-6)], 61.3, 61.4, 61.5, 61.6, 61.7, [6C, 6 x OCH_3(C-3)], 70.8, 70.9, 71.0, 71.06, 71.09, 71.2 (7C, 7 x C-5), 71.36, 71.42, 71.5, 71.78, 71.79 (7C, 7 x C-6), 73.1 ($O\underline{C}H_2CH_2CH_2$-Ph), 79.6, 79.8, 80.2, 80.3, 80.6, 80.8, 81.7, 81.77, 81.83, 81.9, 82.0, 82.1, 82.2, 82.3, 82.4 (21 C , 7 x C-2, 7 x C-3, 7 x C-4), 90.4 (C-I), 98.7, 98.9, 98.96, 99.02, 99.1 (1C, 7 x C-1), 131.1 (CH-Ph), 132.6 (CH-Ph), 141.0 (CH-Ph), 142.7 (2C, C-Ph), 169.5

($\underline{C}$OOH). **HRMS** calcd for $C_{72}H_{119}O_{37}NaI^+$: 1725.6373 $[M+Na]^+$, found: 1725.6339. **HRMS** calcd for $C_{72}H_{118}O_{37}I^-$: 1701.6397 $[M]^-$, found: 1701.6382. . **Melting Point**: >260°C.

2,6-Di-O-methyl-3-O-({3-carboxy-4-iodosophenyl}-propyl)-hexakis-(2,3,6-tri-O-methyl)cyclomaltoheptaose (4): Compound **16** (110 mg, 64.5 µmol) was dissolved in acetic acid (50%, v/v, 2 mL). Then, sodium periodate (1.37 g, 6.4 mmol) was added and the mixture was stirred 24 hours at 45 °C. After cooling to room temperature, the reaction mixture was diluted with water (5 mL) and extracted with dichloromethane (3 × 20 mL). The organic layer was dried over sodium sulfate. After filtration, the solvent was removed under reduced pressure. The residue was purified by reversed phase chromatography on silica gel C18 (ammonium bicarbonate 0.25 M/acetonitrile, 100/0 to 65/35, v/v) to give the desired product (80 mg, 46.5 µmol) after freeze-drying as a white foam. Yield 72%. **^{1}H NMR (300 MHz, $CDCl_3$)**: δ 1.89 – 2.09 (m, 2H, $OCH_2C\underline{H_2}CH_2$-Ph), 2.82 – 3.08 (m, 2H, $OCH_2CH_2C\underline{H_2}$-Ph), 3.19 (m, 7H, 7 x H-2), 3.29 – 3.44 (m, 21H, 7 x $OCH_{3(6)}$), 3.44 – 3.92 (m, 74H, 13 x H-6, 1 x $OC\underline{H_2}CH_2CH_2$-Ph, 7 x H-5, 7 x H-4, 7 x H-3,7 x $OCH_{3(2)}$, 6 x $OCH_{3(3)}$), 3.93 – 4.05 (m, 2H, H-6, 1 x $OC\underline{H_2}CH_2CH_2$-Ph), 5.04 – 5.20 (m, 8H, 7 x H-1, COO$\underline{H}$), 7.73 – 7.88 (m, 2H, 2 x H-Ph), 8.10 (s, 1H, H-Ph). **^{13}C NMR (75 MHz, $CDCl_3$)**: δ 31.8 ($OCH_2CH_2\underline{C}H_2$-Ph), 32.0 ($OCH_2\underline{C}H_2CH_2$-Ph), 58.5, 58.57, 58.60, 58.85, 58.88, 59.0, 59.05, 59.09 [14C, 7 x OCH_3(C-2), 7 x OCH_3(C-6)], 61.2, 61.36, 61.41, 61.49, 61.55, 61.59 [6C, 6 x OCH_3(C-3)], 70.9, 71.0, 71.1, 71.2 (7C, 7 x C-5), 71.4, 71.56, 71.60 (7C, 7 x C-6), 72.8 ($O\underline{C}H_2CH_2CH_2$-Ph), 79.8, 80.0, 80.1, 80.2, 80.4, 80.5, 81.7, 81.8, 81.9, 82.02, 82.06, 82.13, (21C, 7 x C-2, 7 x C-3, 7 x C-4), 98.5, 99.0 (7C, 7 x C-1), 116.73 (C-IO), 126.1 (CH-Ph), 130.4 (C-Ph), 132.2 (CH-Ph), 135.5 (CH-Ph), 146.4 (C-Ph), 170.0 ($\underline{C}$OOH). **HRMS** calcd for $C_{72}H_{119}O_{38}NaI^+$: 1741.6322 $[M+Na]^+$, found: 1741,6337. **HRMS** calcd for $C_{72}H_{118}O_{38}I^-$: 1717.6346 $[M]^-$, found: 1717.6328. **HRMS** calcd for $C_{72}H_{119}O_{38}ClI^-$: 1753.6113 $[M+Cl]^-$, found: 1753.6107. **Melting Point**: >260°C.

2.2.2 Synthesis of compound 5

2-O-({1-Trityl-1H-imidazol-4-yl}methyl)-3,6-di-O-methyl-hexakis-(2,3,6-tri-O-methyl)cyclo-maltoheptaose (18): NaH (60% in mineral oil, 50 mg, 1.235 mmol) was added to a solution of $3^A,6^A$-di-*O*-methylhexakis-(2,3,6-tri-*O*-methyl)cyclomaltoheptaose (**17**, 350 mg, 247 µmol) and 4-(chloromethyl)-1-(triphenylmethyl)imidazole (266 mg, 741 µmol) in 6 mL of anhydrous dimethyl sulfoxide at room temperature under argon atmosphere. The reaction mixture was stirred at room temperature for 16 hours. Then water (30 mL) was added dropwise to quench the reaction. The mixture was extracted with ethyl acetate (3 × 40 mL), the organic layer was washed with brine (100 mL) and dried over sodium sulfate. After filtration the solvent was

removed under reduced pressure. The residue was purified by chromatography on silica gel (cyclohexane/acetone/triethylamine, 70/30/0.5 to 65/35/0.5, v/v/v) to give the desired product (379 mg, 218 µmol) as a white foam. Yield 88% yield. **^{1}H NMR (300 MHz, CDCl$_3$)**: δ 3.06 (dd, ^{3}J = 9.6 Hz, ^{3}J = 3.0 Hz, 1H, H-2), 3.11 – 3.21 (m, 5H, 5 x H-2), 3.28 – 3.43 (m, 23H, 1 x H-2, 1 x H-3, 7 x $OCH_{3(6)}$), 3.43 – 3.67 (m, 59H, 7 x H-4, 7 x H-6, 6 x H-3, 7 x $OCH_{3(3)}$, 6 x $OCH_{3(2)}$), 3.67 – 3.93 (m, 14H, 7 x H-5, 7 x H-6), 4.57 (d, ^{2}J = 12.3 Hz, 1H, 1 x OCH_2-Im), 4.71 (d, ^{2}J = 12.3 Hz, 1H,, 1H, 1 x OCH_2-Im), 4.97 (d, ^{3}J = 2.4 Hz, 1H, H-1), 5.06 – 5.14 (m, 6H, 6 x H-1), 6.89 (s, 1H, 1 x H-Im), 7.06 – 7.15 (m, 5H,5 x H-Ph_{Tr}), 7.24 – 7.34 (m, 10H,10 x H-Ph_{Tr}), 7.36 (s, 1H,1 x H-Im). **^{13}C NMR (75 MHz, CDCl$_3$)**: δ 58.5, 58.57, 58.62, 58.7, 58.9, 59.02, 59.04, [13C, 6 x OCH_3(C-2), 7 x OCH_3(C-6)], 61.3, 61.4, 61.50, 61.54, 61.57 [7C, 7 x OCH_3(C-3)], 67.0 (OCH_2-Im), 70.9, 71.0, 71.2 (7C, 7 x C-5), 71.36, 71.44, 71.5, 71.6, 71.7 (7C, 7 x C-6), 75.4 (C_{IV} trityl), 79.7, 80.0, 80.2, 80.26, 80.34, 80.5, 80.6, 81.75, 81.78, 81.9, 82.0, 82.11, 82.2 (21C, 7 x C-2, 7 x C-3, 7 x C-4), 99.1, 99.4(7C, 7 x C-1), 120.36 (CH-Im), 128.1, 129.9 (15C, CH-Ph_{Tr}), 138.5 (CH-Im), 139.0 (C-Im), 142.6 (3C, C-Ph_{Tr}). **HRMS** calcd for $C_{85}H_{128}N_2O_{35}Na^+$: 1759.8195 [M+Na]$^+$, found: 1759.8162. **Melting Point**: >260°C.

2-O-({1H-Imidazol-4-yl}methyl)-3,6-di-O-methyl-hexakis-(2,3,6-tri-O-methyl)cyclomalto-heptaose (5): Compound **18** (100 mg, 57.5 µmol) was dissolved in acetic acid (50%, v/v, 2 mL) and the solution was stirred 14 hours at 45 °C. After cooling to room temperature an aqueous solution of sodium hydroxide (60 mL, 0.5 M) was added to the reaction mixture. The solution was extracted with dichloromethane (2 × 25 mL), the organic layer washed with brine (50 mL), and dried over sodium sulfate. After filtration, the solvent was removed under reduced pressure. The residue was purified by chromatography on silica gel (dichloromethane/methanol/triethylamine, 95/5/0.5 to 90/10/0.5, v/v/v) to give the desired product (81 mg, 54.2 µmol) as a white foam. Yield 94%. **^{1}H NMR (300 MHz, CDCl$_3$)**: δ 3.07 – 3.23 (m, 6H, 6 x H-2), 3.25 – 3.40 (m, 22H, 1 x H-2, 7 x $OCH_{3(6)}$), 3.40 – 3.67 (m, 60H, 7 x H-4, 7 x H-6, 7 x H-3, 7 x $OCH_{3(3)}$, 6 x $OCH_{3(2)}$), 3.67 – 3.91 (m, 14H, 7 x H-5, 7 x H-6), 4.59 (d, ^{2}J = 12.5 Hz, 1H, 1 x C<u>H</u>$_2$-Im), 4.71 (d, ^{3}J = 2.6 Hz, 1H, 1 x H-1), 4.76 (d, ^{2}J = 12.5 Hz, 1H, 1 x C<u>H</u>$_2$-Im), 5.13 – 5.05 (m, 5H, 5 x H-1), 5.14 (d, ^{3}J = 3.1 Hz, 1H, 1 x H-1), 6.90 (s, 1H, H-Im), 7.53 (s, 1H, H-Im). **^{13}C NMR (75 MHz, CDCl$_3$)**: δ 58.42, 58.55, 58.58, 58.8, 58.96, 59.00, 59.02, 59.03, 59.1 [13C, 6 x OCH_3(C-2), 7 x OCH_3(C-6)], 61.3, 61.4, 61.5, 61.6, 61.8, [7C, 7 x OCH_3(C-3)], 64.5 (OCH_2-Im), 70.7 (1C, 1 x C-6), 70.8, 70.9, 71.0, 71.1, 71.2 (7C, 7 x C-5), 71.4, 71.5, 71.6 (6C, 6 x C-6), 80.1, 80.2, 80.3, 80.4, 80.47, 80.53, 81.8, 81.9, 82.0, 82.06, 82.12, 82.25, 82.28, 82.4 (21C, 7 x C-2, 7 x C-3, 7 x C-4), 98.89, 98.92, 99.1, 99.36, 99.43 (7C, 7 x C-1), 124.6 (broad signal, C<u>C</u>HN-Im), 129.2 (broad signal, C-Im), 135.6

(NHCHN-Im). **HRMS ESI**+ calcd for $C_{66}H_{115}N_2O_{35}^+$: 1495.7280 [M+H]$^+$ found: 1495.7263. **HRMS ESI**+ calcd for $C_{66}H_{114}N_2O_{35}Na^+$: 1517.7100 [M+Na]$^+$, found:1517.7076. **Melting Point**: >260°C.

3. ^{1}H NMR and ^{13}C NMR spectra of compounds 13, 14, 16, 18

2^{B}-O-({1-Trityl-1H-imidazol-4-yl}methyl)-3^{A}-O-(3-carboxypropyl-4-iodobenzyl)-2^{A},3^{B},6^{A},6^{B}-tetra-O-methyl-pentakis-(2,3,6-tri-O-methyl)cyclomaltoheptaose (13)

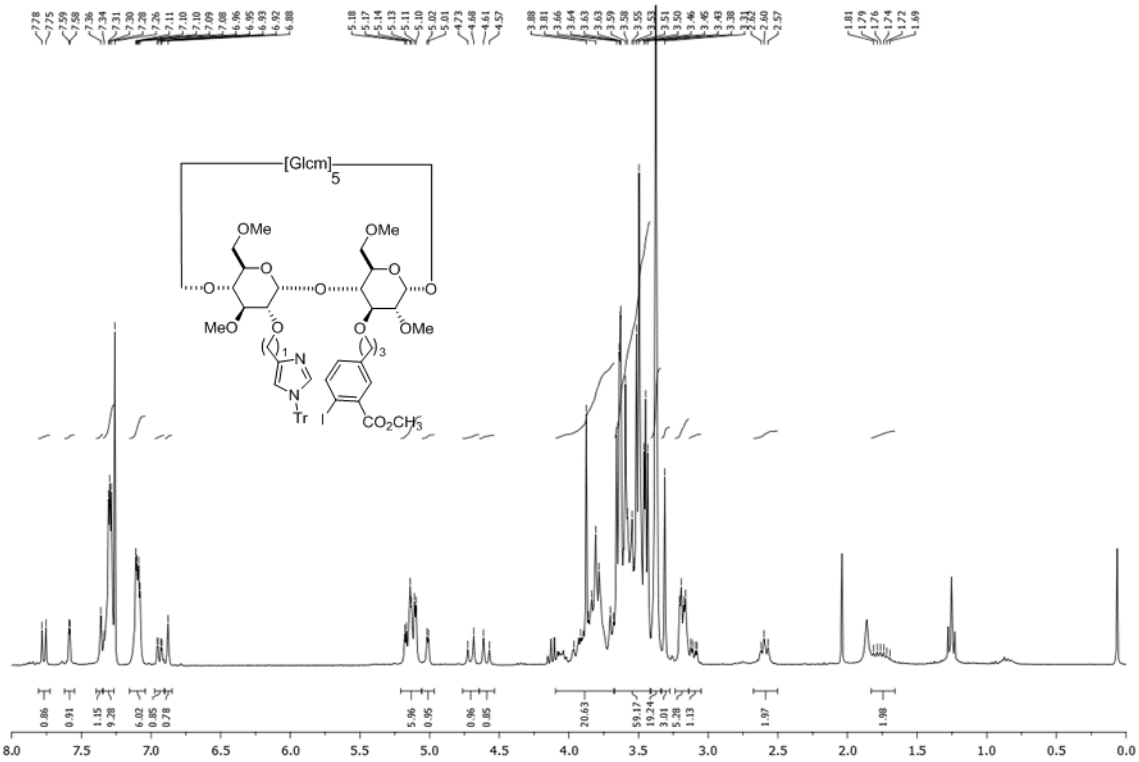

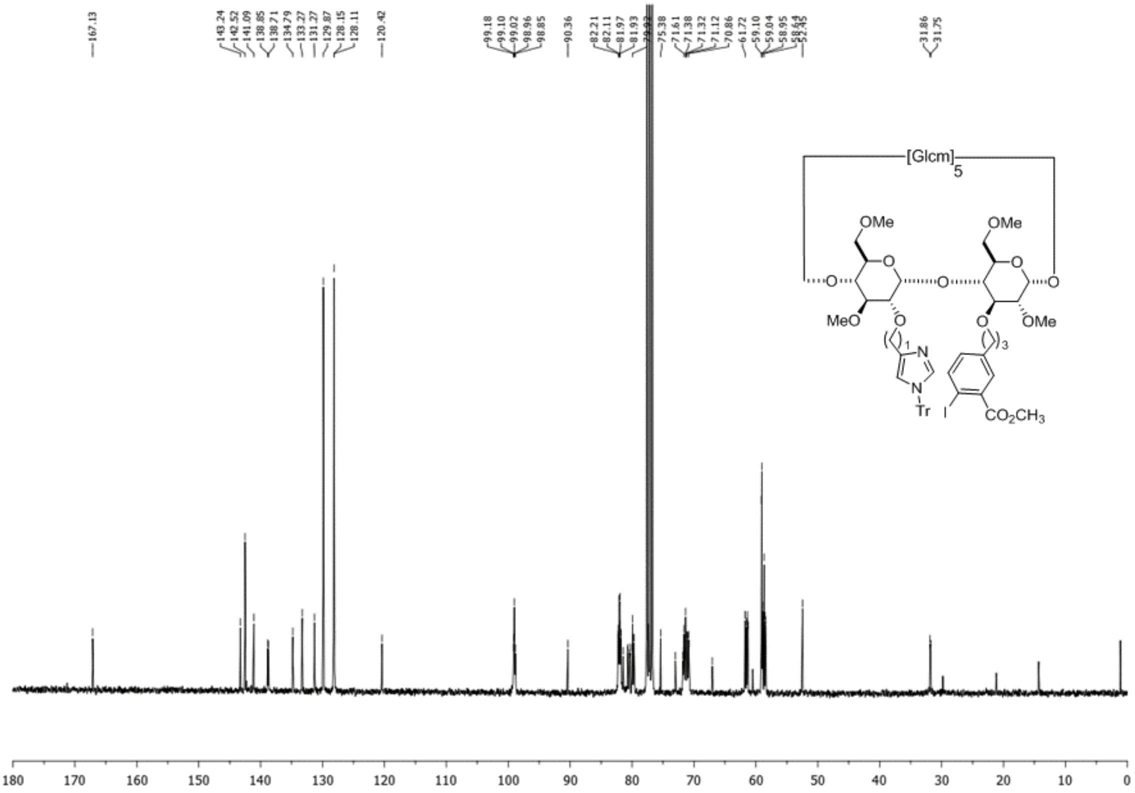

2^{B}-O-({1-Trityl-1H-imidazol-4-yl}propyl)-3^{A}-O-(3-carboxymethyl-4-iodobenzyl)-2^{A},3^{B},6^{A},6^{B}-tetra-O-methyl-pentakis-(2,3,6-tri-O-methyl)cyclomaltoheptaose (14)

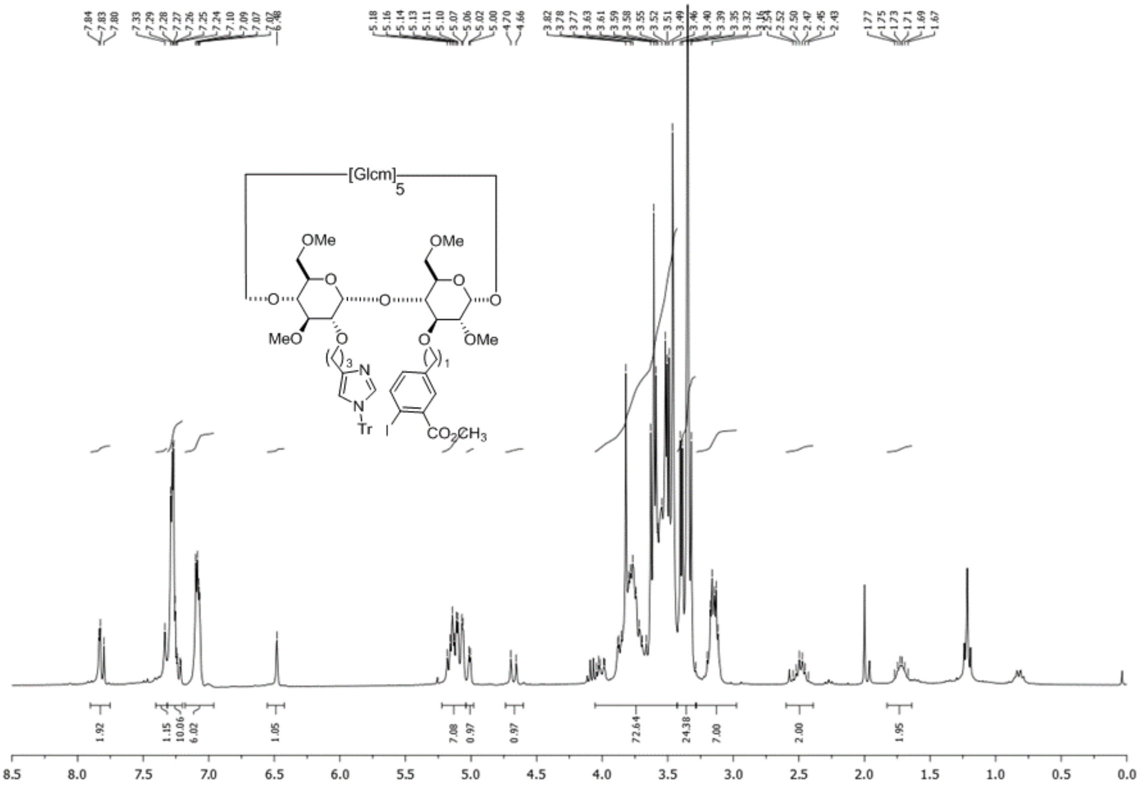

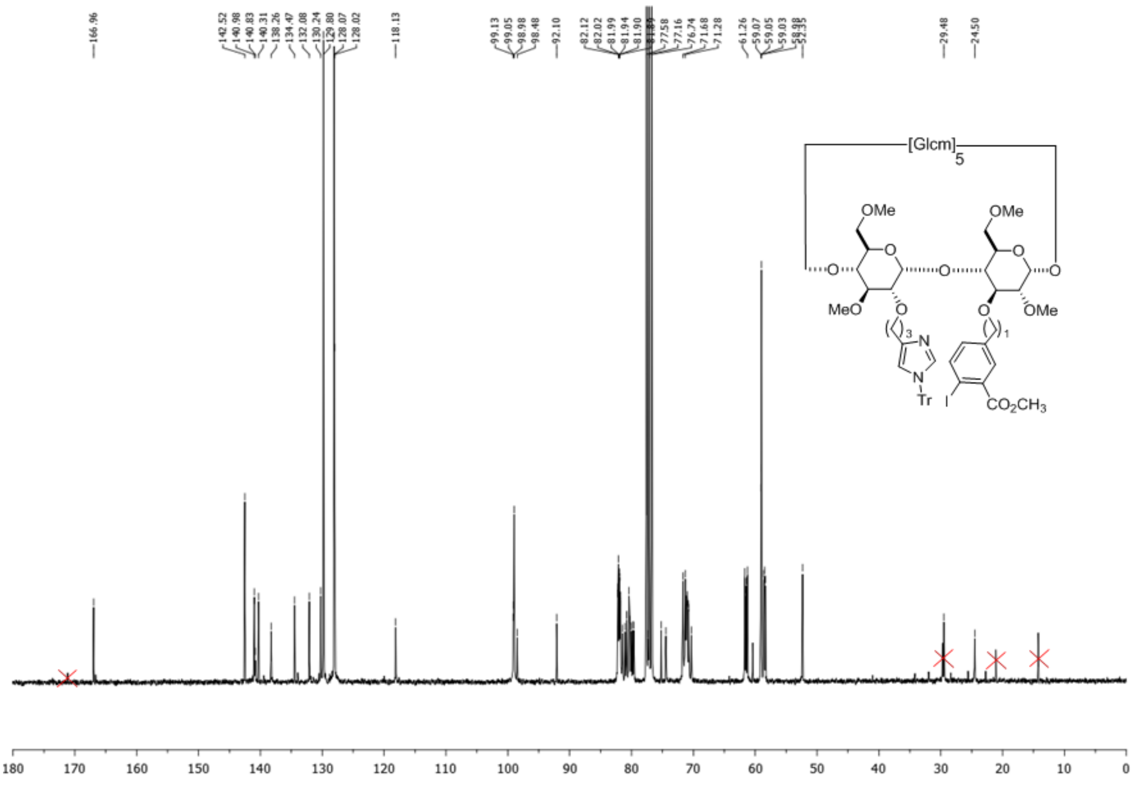

2,6-Di-O-methyl-3-O-({3-carboxy-4-iodophenyl}-propyl)-hexakis-(2,3,6-tri-O-methyl)cyclomaltoheptaose (16)

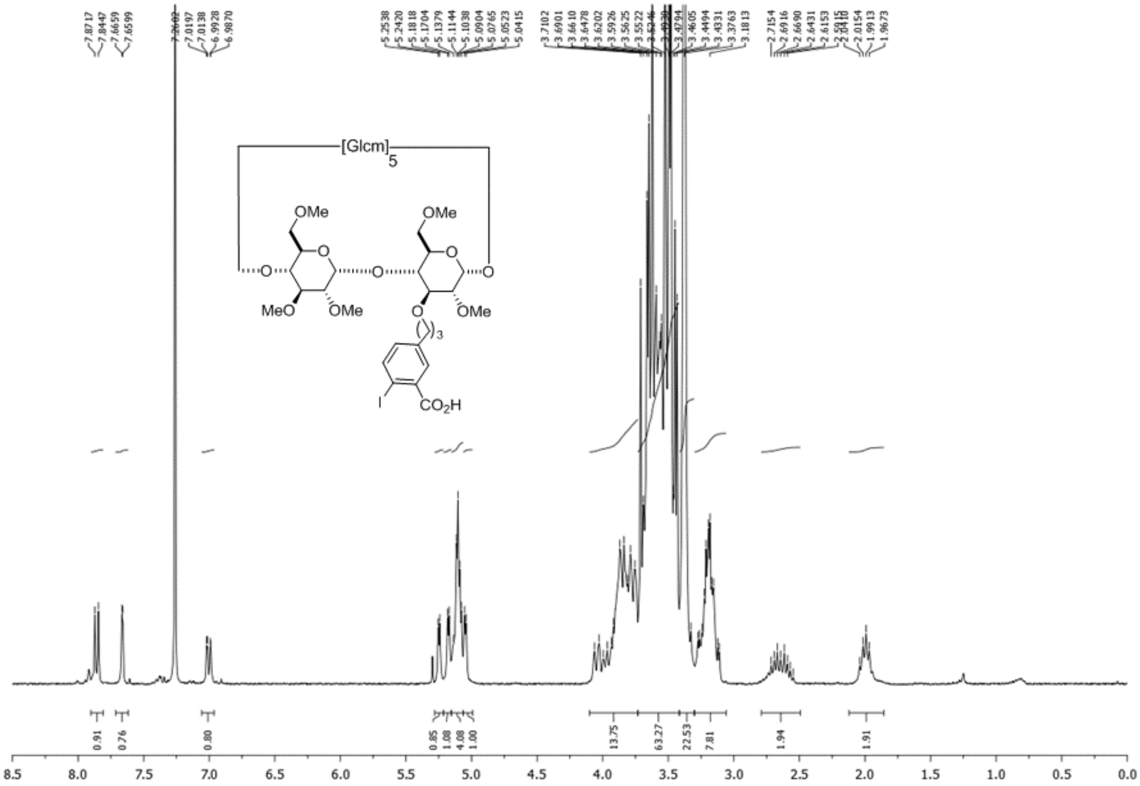

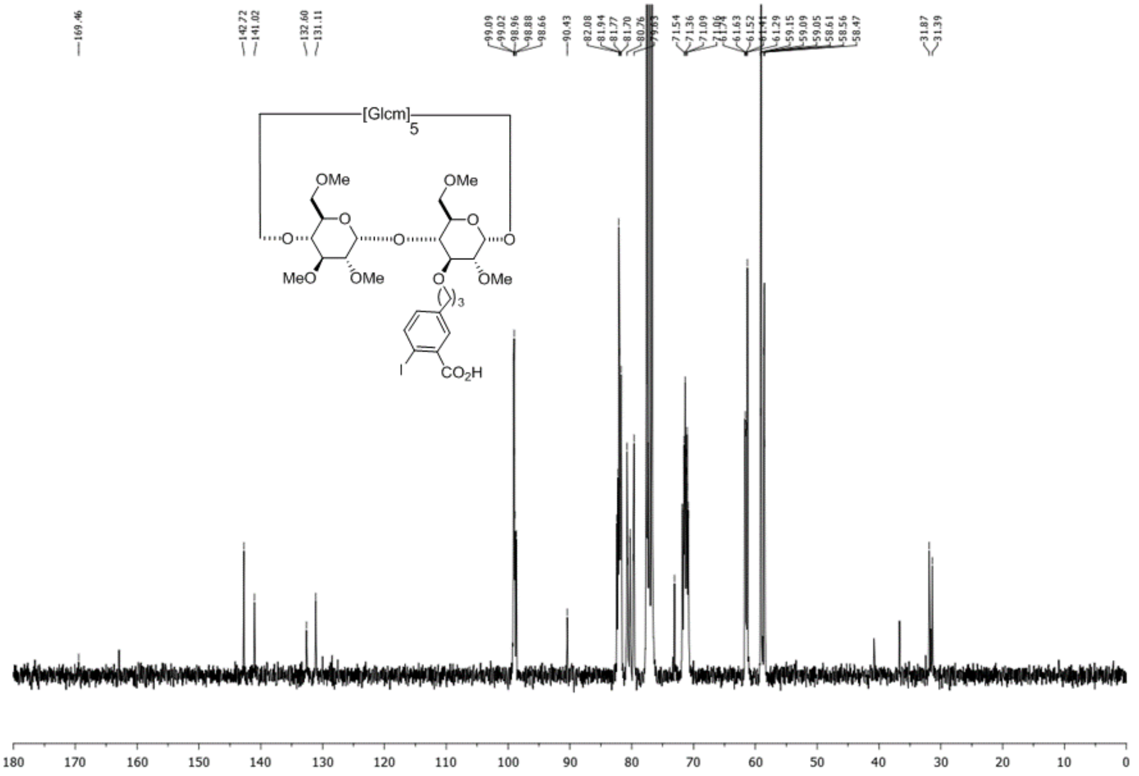

2,6-Di-O-methyl-3-O-({3-carboxy-4-iodophenyl}-propyl)-hexakis-(2,3,6-tri-O-methyl)cyclomaltoheptaose (16)

Partial COSY spectrum

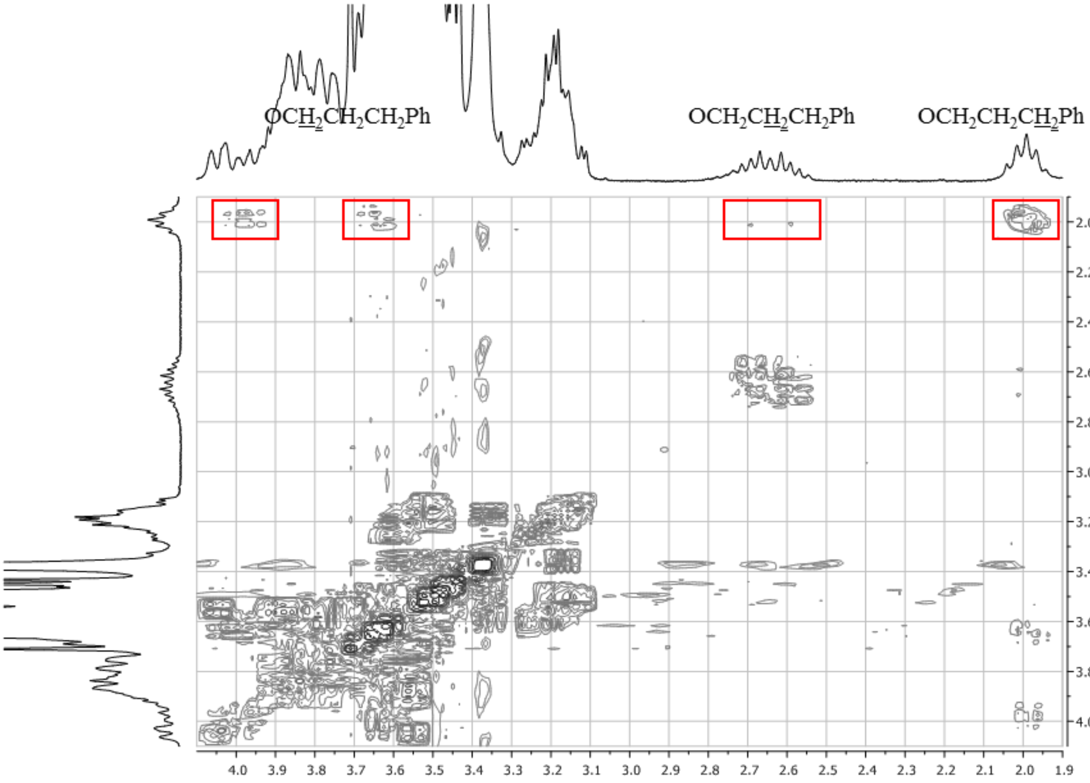

Partial HSQC spectrum

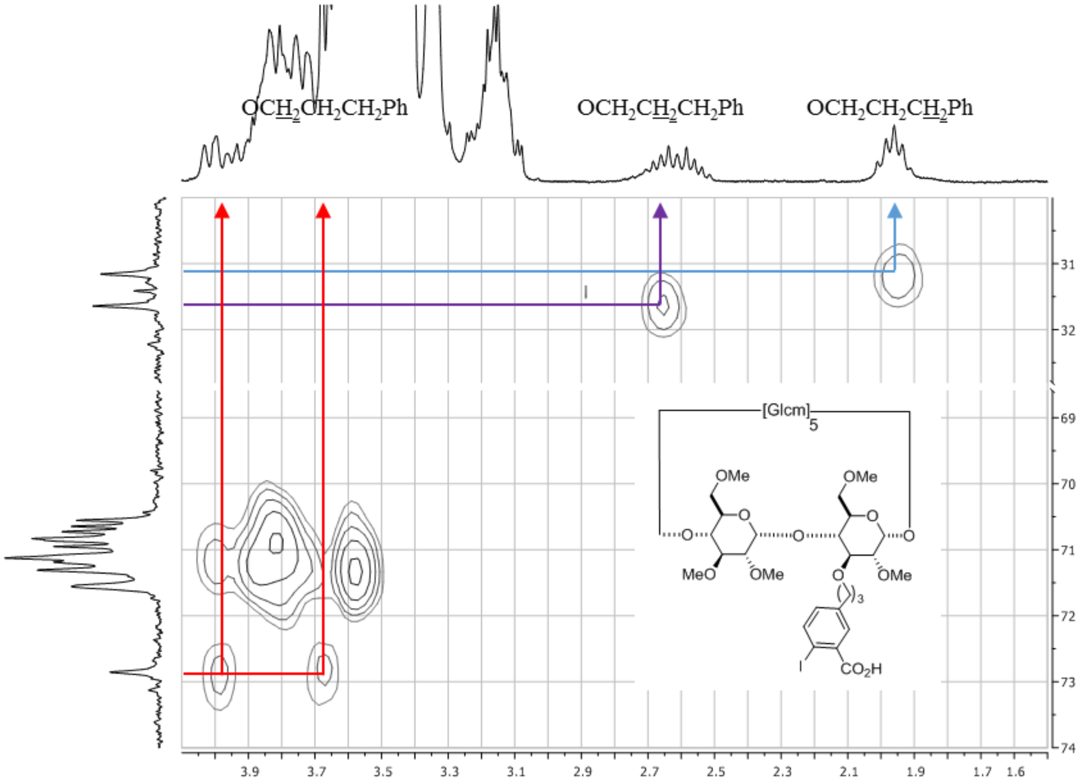

2-O-({1-Trityl-1H-imidazol-4-yl}methyl)-3,6-di-O-methyl-hexakis-(2,3,6-tri-O-methyl)cyclomaltoheptaose (18)

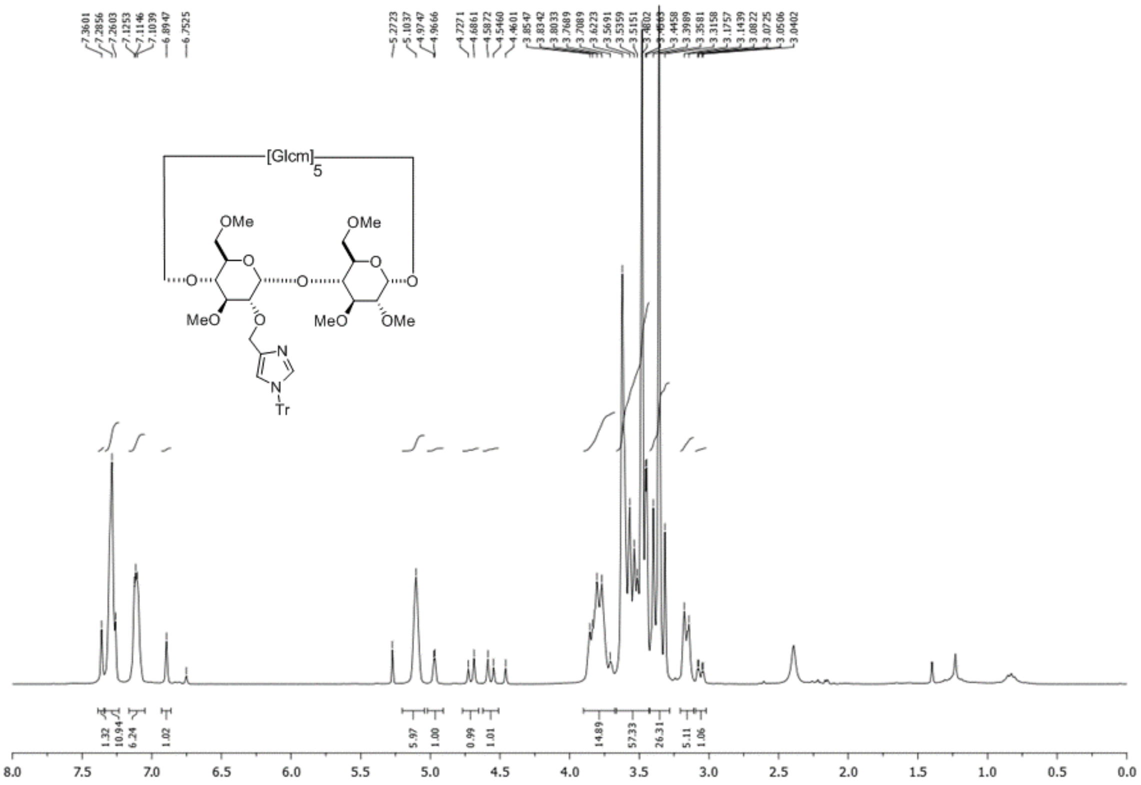

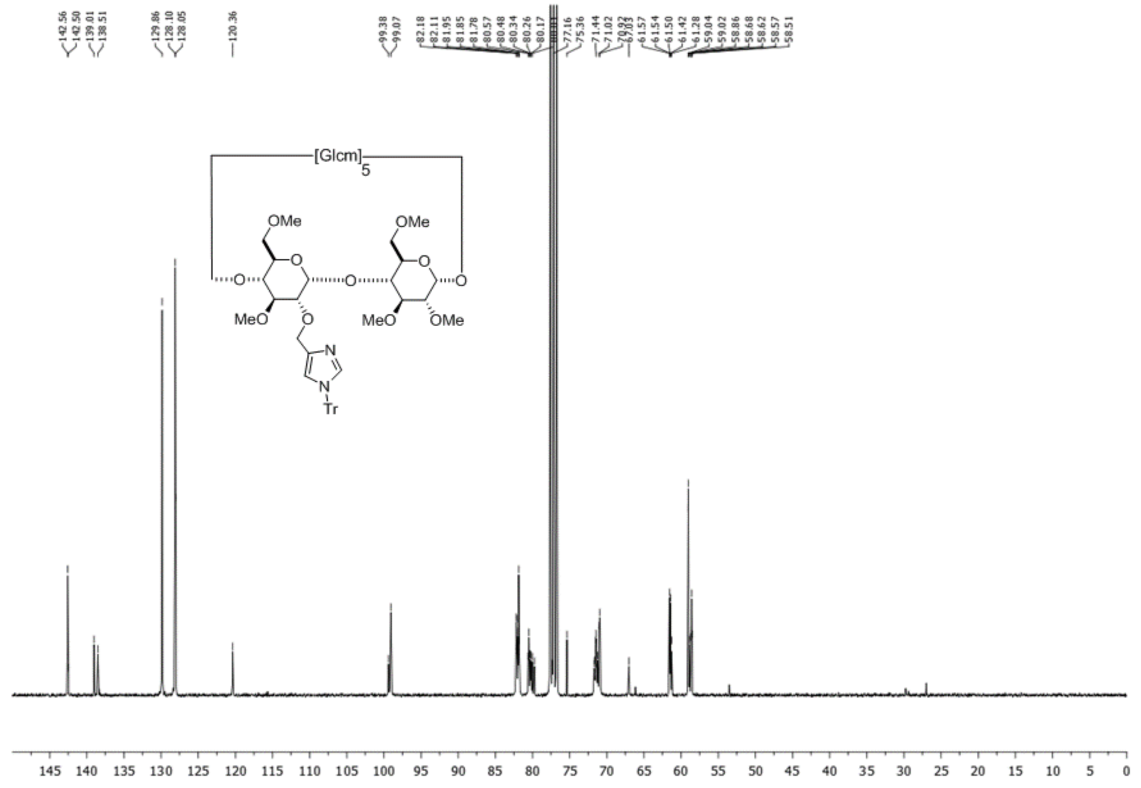

4. Hydrolytic assays of pesticides

4.1 Preparation of the stock solutions

Methyl paraoxon, methyl parathion, and fenitrothion were the organophosphates used for the kinetics assays: Stock solutions were prepared at a concentration of 16.67 mM in anhydrous methanol.

Stock solutions of β-CD derivatives **2**, **3**, **4**, **5**, 2-iodosobenzoic acid (IBA), imidazole, and TRIMEB (2.5 mM) were prepared in a mixture of 3% v/v DMSO in 20.62 mM sodium phosphate buffer pH 7.65 in the presence of 13.4 mM cetyltrimethylammonium chloride (CTAC).

4.2 Kinetic assays

Kinetic assays were carried out with methyl paraoxon, methyl parathion or fenitrothion (0.5 mM) at 25 °C, in 20 mM sodium phosphate buffer pH 7.65 in the presence of cetyltrimethylammonium chloride (13 mM). The final concentration of methanol in the assay was 3% v/v and that of DMSO was 2.9% v/v. The final concentrations of β-CD derivatives **2**, **3**, **4**, **5**, 2-iodosobenzoic acid (IBA), imidazole, and TRIMEB were 0.5 mM or 0.25 mM. The hydrolysis of organophosphates was monitored up to 10 min by following the release of the leaving group *p*-nitrophenol (at λ = 400 nm). From the measured absorbance, the absorbance due to spontaneous hydrolysis of the organophosphates was subtracted. The spontaneous hydrolysis was measured with a cuvette containing the organophosphate (0.5 mM), methanol (3% v/v), DMSO (2.9%, v/v) 20 mM phosphate buffer pH 7.65 in the presence of cetyltrimethylammonium chloride (13 mM). Each experiment was conducted in triplicate.

4.3 UV–vis calibration curves

UV–vis calibration curves were carried out with solutions of 4-nitrophenol (at concentrations ranging from 0.005 mM to 0.1 mM) in 20 mM phosphate buffer pH 7.65, 13 mM CTAC, 2.9 vol % DMSO, 3 vol % CH_3OH at 25 °C. The absorbance of 4-nitrophenol was not modified by the presence of TRIMEB (0.25 mM), compound **2** (0.25 mM) or compound **4** (0.25 mM). The calibration curves were recorded in triplicate.

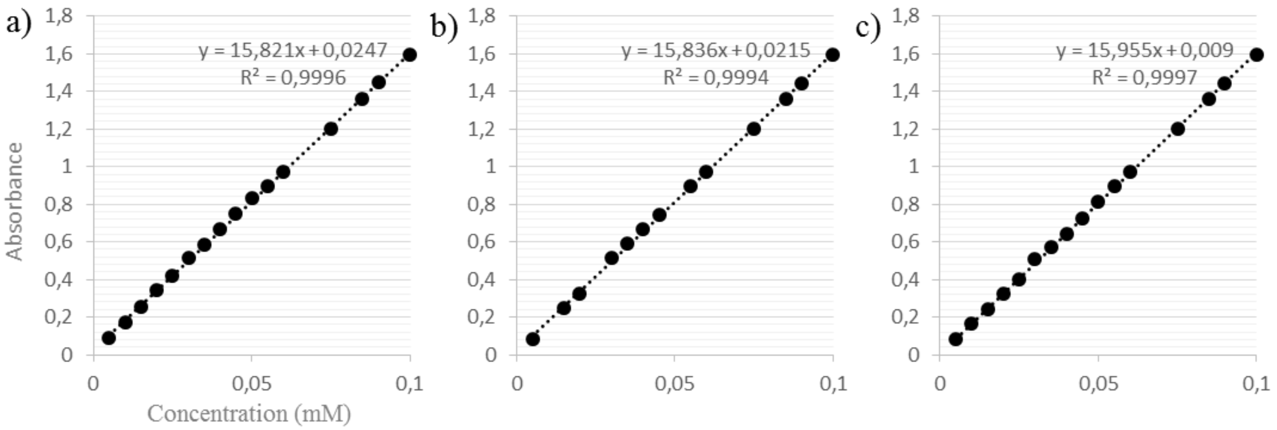

UV–vis calibration curves of 4-nitrophenol at λ = 400 nm, 25 °C.

The correlation of the measured absorbance values to the 4-nitrophenol calibration curves were used to determine the pseudo-first-order rate constants of the hydrolyses of the pesticides (methyl paraoxon and methyl parathion).

5. Detoxification assays of soman

Soman (GD, 500 nM) was incubated with the cyclodextrin derivatives **1**, **2**, **3**, or **4** (250 µM) in phosphate buffer (0.1 M) at pH 7 and 37 °C for up to 60 min. At different time points (10, 20, 30, 40, 50 and 60 min), samples (20 µL of the solution) were taken and incubated with 180 µL of AChE (activity was adjusted to 1 U/mL) for 3 min at 37 °C. Subseqently, 50 µL of Ellman solution were added and the AChE activity was measured spectrophotometrically at 412 nm.

The experiments were performed in quadruplicate and data are the means ± SD of four experiments.
